# Frictional Dissipation and Scaling Laws at van der Waals Interfaces: The Role of Edge and Corner Elastic Moiré Pinning

**DOI:** 10.1021/acsnano.5c04617

**Published:** 2025-08-11

**Authors:** Xiang Gao, Weidong Yan, Wengen Ouyang, Ze Liu, Michael Urbakh, Oded Hod

**Affiliations:** † CAS Key Laboratory of Mechanical Behavior and Design of Materials, Department of Modern Mechanics, University of Science and Technology of China, Hefei, Anhui 230027, China; ‡ Department of Engineering Mechanics, School of Civil Engineering, Wuhan University, Wuhan, Hubei 430072, China; § State Key Laboratory of Water Resources Engineering and Management, Wuhan University, Wuhan, Hubei 430072, China; ∥ Department of Physical Chemistry, School of Chemistry, The Raymond and Beverly Sackler Faculty of Exact Sciences and The Sackler Center for Computational Molecular and Materials Science, Tel Aviv University, Tel Aviv 6997801, Israel

**Keywords:** van der Waals interface, structural superlubricity, energy dissipation, scaling laws, kinetic friction, moiré pinning

## Abstract

van der Waals heterogeneous interfaces are promising candidates for the scaling-up of structural superlubricity to meet a wide range of applications. Several factors, however, have been identified that may hinder superlubricity. Elasticity is one such intrinsic factor, where shear induced lattice reconstruction leads to local interfacial pinning, even at clean pristine contacts. This introduces intricate energy dissipation mechanisms that are manifested by unconventional frictional scaling laws. Here, through large-scale atomistic simulations, we reveal that the elastic pinning of incomplete moiré tiles at the corners of polygonal sliders dominates kinetic friction up to contact dimensions of hundreds of nanometers, followed by a crossover to edge, and eventually surface dominated frictional regimes. We further demonstrate that slider shape tailoring and twisting allow to control energy dissipation and its scaling with contact size, thus advancing the quest toward achieving large-scale superlubricity.

## Graphical abstract

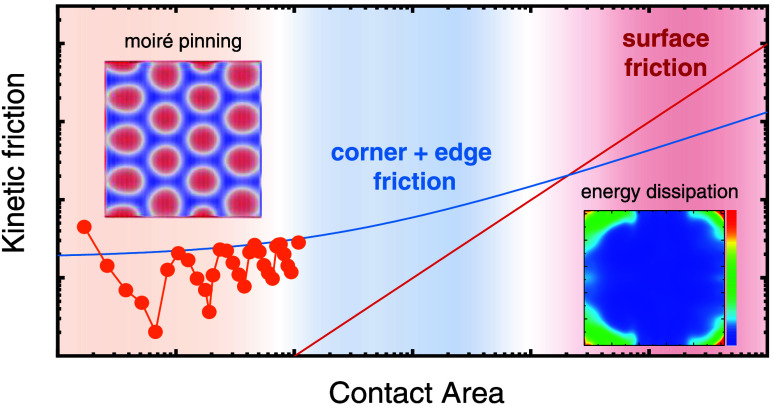

## Introduction

Structural superlubricity (SSL), the intriguing phenomenon of ultralow friction and wear occurring at incommensurate solid–solid flat crystalline interfaces, provides vast opportunities for the reduction of energy dissipation and wear in mechanical systems at various length scales.^1^ This unique phenomenon was first demonstrated experimentally for twisted graphitic nanocontacts and later extended to incommensurate microscale junctions.^2,3^ However, for such homogeneous contacts, SSL is prone to dynamical reorientation of the slider into the commensurate low energy and high-friction configuration.^4^ Conversely, van der Waals (vdW) heterostructures, such as graphene (Gr)/hexagonal boron nitride (*h*-BN), Gr/molybdenum disulfide (MoS_2_), and *h*-BN/MoS_2_, present an intrinsic lattice mismatch (∼1.8% for Gr/*h*-BN, ∼26.8% for Gr/MoS_2_, and ∼24.6% for *h*-BN/MoS_2_), such that even the aligned configuration remains incommensurate. This, in turn, prevents interlocking of the interface into a high friction state, leading to superlubric behavior that is robust against interfacial reorientation.^5,6^ First predicted theoretically,^5^ this effect was recently demonstrated experimentally for microscale contacts even under ambient conditions.^7,8^

The scaling-up of robust SSL, however, results in the emergence of new energy dissipation mechanisms including interfacial elasticity effects,^9–14^ grain boundary dynamics,^15–20^ edge and surface lattice defect reactivity,^21–25^ and contaminant intercalation.^26–30^ The former, is often associated with bulk frictional contributions, which are expected to appear at the micro- and macro-scales. Atomistic simulations of interfacial elasticity effects at such scales, however, are often limited by computational complexity. Nonetheless, edge elasticity effects may kick-in already at smaller contact dimensions. Hence, it is desirable to formulate general scaling laws that can extrapolate information gained from smaller-scale junction model simulations toward the understanding of macroscale elasticity effects.

While experiments on layered material contact sliding measure their dynamical friction properties that inherently involve edge and surface elasticity effects,^8,21,22,31–36^ previous computational studies focused mainly on the static friction characteristics of rigid finite layered contacts.^37–40^ Kinetic friction properties have been studied, but only for circularly shaped sliders.^11^ Of more relevance to experiments, however, would be the study of the kinetic friction of the abundant polygonal shaped sliders.^3,7,8,19^ To that end, in this paper we present the results of large-scale (up to hundreds of nanometers in size) fully atomistic simulations of flexible multilayer Gr/*h*-BN interfaces of various polygonal contact geometries. We find that elastic pinning of incomplete moiré tiles at the edges and corners of the slider plays a key role in energy dissipation in aligned and marginally twisted layered interfaces. This leads to distinct periodicity and shape dependence of the kinetic friction scaling laws. We further identify a sublinear-to-linear transition of the kinetic friction scaling with contact dimensions when surface moiré superstructure dynamics becomes dominant.

## Results and Discussion

### Simulation Setup

Our model system for Gr/*h*-BN heterojunctions, shown in Figure 1a, contains a three-layer-thick finite sized ABA- (Bernal-) stacked Gr flake that is hydrogen-saturated at its rim atoms, residing atop a three-layer laterally periodic AA’A-stacked *h*-BN substrate. To avoid spurious interactions between periodic flake replicas, the lateral dimensions of the *h*-BN substrate are chosen to be ∼10 nm larger than those of the Gr flake. As shown in Figure 1b, the top Gr layer is set to be rigid and is driven along the armchair (*x*) direction of the substrate with a constant velocity of *v*_0_ = 5 m/s. The bottom *h-*BN layer is frozen at its initial position. To evaluate the effect of contact geometry, we consider four regular polygonal flake shapes, i.e., triangular, square, rectangular, and hexagonal. The geometric center of the Gr flake is initially positioned such that it crosses B and N atoms when sliding along a scanline that is directed in the armchair axis of the *h*-BN substrate. In this way, the emerging moiré superstructures (see Figure 1c) that include commensurate regions (red) and elevated ridges (blue), present mirror symmetry with respect to the line crossing the center of the flake parallel to the sliding (*x*-axis) direction. Notably, due to the finite size of the flake, incomplete moiré tiles appear at the edges, where enhanced flexibility allows for larger out-of-plane deformation toward the substrate.

**Figure 1:**
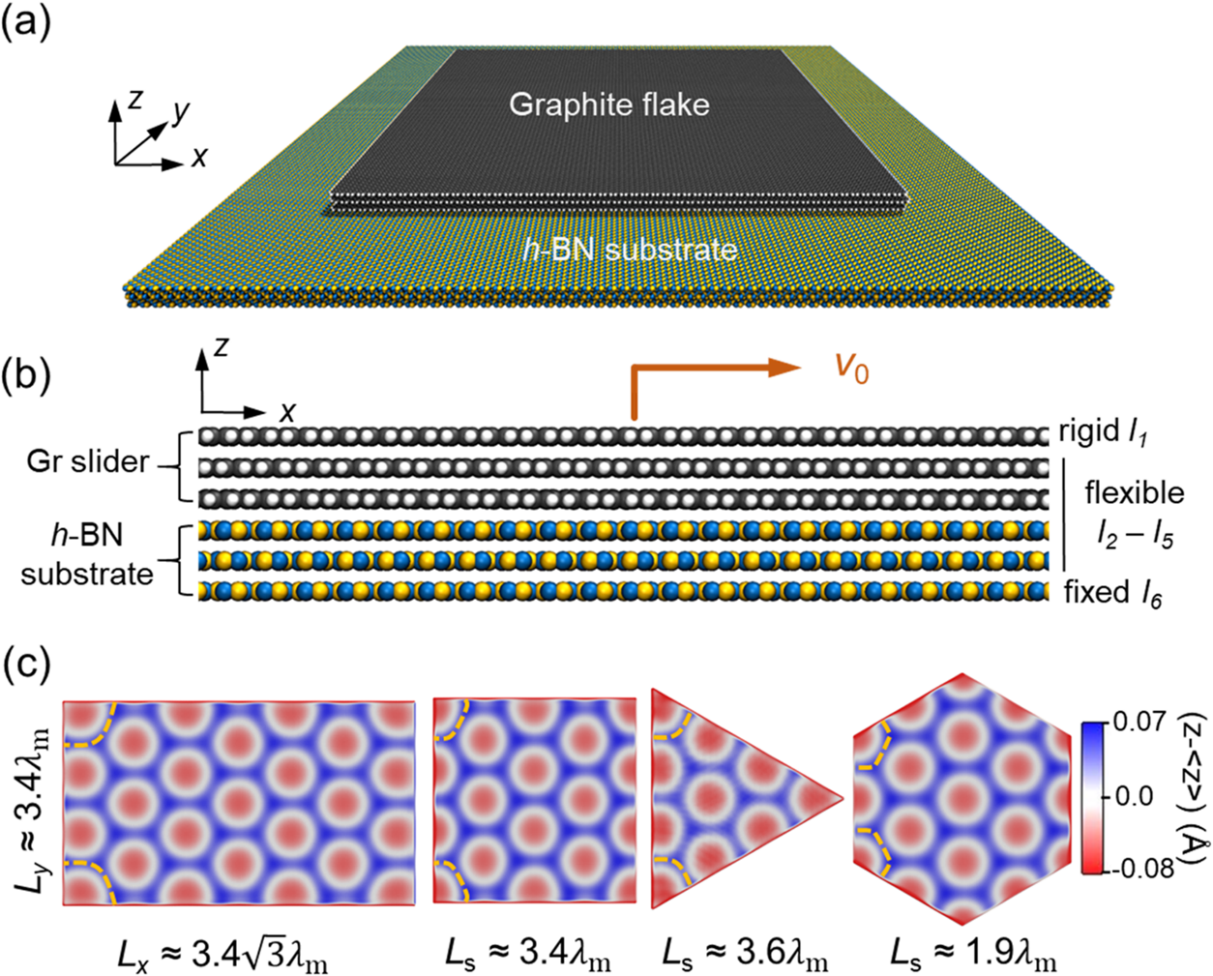
Model system setup. (a) Perspective view of a representative Gr/*h*-BN heterostructure. From top to bottom, the system consists of a three-layer thick Gr slider with hydrogen-saturated rim atoms (gray and white spheres, respectively) atop a three-layer periodic *h*-BN substrate (blue and yellow spheres, respectively). (b) Side view of the Gr/*h*-BN heterostructure. (c) Representative color maps of the topographies of the relaxed interfacial Gr layer (l_3_) for four geometries signifying the emerging moiré superstructures. In the scale bar, *z* represents the atomic height and ⟨*z*⟩ is the average height. *L*_*x*_, *L*_*y*_, and *L*_*s*_ denote *x*- and *y*-axis side length of the rectangular flake and the side length of all other regular polygons, respectively, and λ_m_ ≈ 13.9 nm is the moiré period in the aligned configuration, which is the maximum that can be achieved for the relaxed system. The yellow dashed arcs mark the incomplete moiré tiles at the left corners of the flakes.

The sliding simulations are performed at zero temperature using damped dynamics as done in previous studies.^15,16^ This enables the clear identification of the dominant energy dissipation channels, while avoiding thermal fluctuation effects. At finite temperature, thermal activation is expected to reduce the overall friction but should not alter the qualitative nature of our simulation results.

We note that the high sliding velocity used in our simulations is typical for atomistic simulations in the field of nanotribology and is a consequence of the computational burden involved with evaluating the forces and propagating the equations of motion. In Supporting Information Section 1, we verify that our main findings are relevant to realistic experimental scenarios of much lower sliding velocities, by repeating some of our simulations both at a lower sliding velocity of 2 m/s and under quasi-static conditions.

Further details regarding the simulation protocol and friction force calculations are provided in the Methods section and in Supporting Sections 1 and 2. Supporting Movie S1 presents a typical sliding simulation of a square flake, where the corner regions exhibit stick–slip motion.

### Static and Kinetic Friction Dependence on the Dimensions of Aligned Polygonal Flakes

We start by examining rectangular flakes, which allow to independently study the frictional dependence on the two lateral flake dimensions, *L*_*x*_ and *L*_*y*_. The flakes are positioned in the aligned configuration, such that two of their parallel edges are perpendicular to the sliding (*x*) direction and parallel to one of the moiré supercell lattice vectors. Figure 2a,b shows the static (*F*_s_, panel a) and the kinetic (*F*_k_, panel b) friction forces as a function of *L*_*x*_ (black circles) or *L*_*y*_ (red diamonds), where the other lateral flake dimension remains fixed. Interestingly, the static and kinetic friction forces demonstrate distinct behaviors. When increasing *L*_*x*_, the static friction exhibits pronounced oscillations about a constant average, whereas the kinetic friction remains nearly independent of the edge size for the contact dimensions considered. Conversely, with growing *L*_*y*_, the static friction increases linearly while its kinetic counterpart exhibits strong oscillations with some apparent overall growing envelope. Notably, the periodicities of the static friction with *L*_*x*_ and the kinetic friction with *L*_*y*_ are determined by the moiré superstructure periodicity along the corresponding direction, such that λ_s_^(*x*)^ ∼ $\sqrt{3}$ λ_m_/2 and λ_k_^(*y*)^ ∼ λ_m_, respectively. This suggests that the superstructure plays an important role in the different size dependencies obtained.

**Figure 2:**
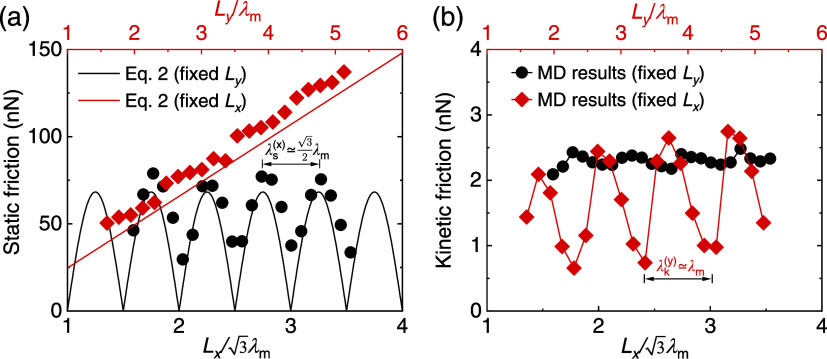
Scaling of the static and kinetic friction forces with the dimensions of rectangular flakes. (a) Static, *F*_s_, and (b) kinetic, *F*_k_, friction forces calculated for rectangular flakes as a function of side length. Black symbols (bottom horizontal axis) and red symbols (upper horizontal axis) correspond to the conditions of a fixed *L*_*y*_ = 2.76 λ_m_ and a fixed *L*_*x*_ = 2.76 $\sqrt{3}$ λ_m_, respectively. The solid lines in panels (a) are theoretical predictions obtained using eq 2.

When keeping the aspect ratio of the rectangular (or square) flake constant, a linear combination of the individual side dependencies discussed above is expected to occur. This can be clearly seen in Figure S3, where the static and kinetic friction forces exhibit oscillations with a linearly growing envelope and periods of λ_s_ ∼ $\sqrt{3}$ λ_m_ and λ_k_ ∼ λ_m_, respectively, for both the rectangular and square flakes when the contact encompasses at least a single moiré supercell. For smaller contact dimensions, edge and corner pinning of the lower graphene layer to the underlying *h*-BN surface result in increased kinetic friction (see Figure S3b) and may induce stacking instability (shifts between the ABA ⇌ ABC stacking modes) or permanent stacking transformation (ABA ⇒ ABC) in the trilayer Gr flake.^6^

A similar behavior is obtained for the triangular flakes, where both the static and the kinetic friction forces exhibit linear scaling with side length, *L*_s_, accompanied by oscillations of period λ_m_ (See Supporting Section 3). Notably, while the qualitative behavior of the kinetic friction force of hexagonal flakes is the same, the static friction force shows oscillations of half the period λ_m_/2 with side length. This reduced periodicity is due to the fact that for a given side length, the hexagonal *x* dimension is twice as that of the triangular one. This affects the mutual compensation of incomplete moiré tiles from different edges.^39^

### Analytical Model for Static Friction

The static friction variation with flake dimensions obtained via the atomistic simulations can be rationalized by an analytical model that is based on rigid flake sliding considerations. For the Gr/*h*-BN interface, the interlayer interactions can be approximated by a continuum potential energy density function of the form^39,51^

1 $$U\left(x,y\right)=-\frac{2}{9}U_{0}\left(2\cos\frac{2\pi x}{\sqrt{3}\lambda_{m}}\cos\frac{2\pi y}{\lambda_{m}}+\cos\frac{4\pi x}{\sqrt{3}\lambda_{m}}\right)$$

where the value of the energy corrugation parameter *U*_0_ = 4.5 meV/Å^2^ is chosen to match density functional theory (DFT) reference data.^52^ We note that this coarse-grained potential map describes moiré supercell scale interactions and does not account for atomistic features. The total interlayer energy of a flake of surface area *A* can be calculated as *E*(*A*, *x*_0_,*y*_0_) = *∫*_*A*(*x*_0_,*y*_0_)_ *U*d*A*, where (*x*_0_, *y*_0_) is the geometric center of the flake with respect to the domain center of one of the moving moiré tiles (the specific identity of the tile is unimportant). The corresponding static friction force can be evaluated as the maximal derivative of the interlayer energy along the scanline (chosen here to be parallel to the *x* axis): $F_{s,\mathrm{rigid}}=\left(\frac{\lambda_{m}}{a_{h\mathrm{BN}}}\right)\max_{x_{0}}\left(-\frac{dE}{dx_{0}}\right)$ , where *a*_*h*BN_ = 2.5045 Å is the lattice period of *h*-BN. The prefactor accounts for the fact that in the coarse-grained potential, all distances are scaled with respect to the moiré supercell dimensions, whereas the derivatives should be taken with respect to the atomic lattice displacements. We note that this analytical model, based on the rigid flake assumption, was shown to provide excellent agreement with atomistic force-field calculations of the static friction of rigid layered contacts.^39^

Using this analytical scheme, the static friction of rectangular shaped flakes with *L*_*y*_ ≫ λ_m_, can be approximated as (see Supporting Section 4)­

2 $$F_{s,\mathrm{rigid}}\approx \frac{4}{9}\frac{\lambda_{m}L_{y}U_{0}}{a_{h\mathrm{BN}}}\left|\sin(\frac{2\pi L_{x}}{\sqrt{3}\lambda_{m}})\right|$$

and its upper envelope reads as

3 $$F_{s,\mathrm{rigid}}^{\mathrm{up}}=\frac{4}{9}\frac{\lambda_{m}L_{y}U_{0}}{a_{h\mathrm{BN}}}$$

Equations 2 and 3 agree well with the MD simulation results for the static friction of the flexible interfaces presented in Figures 2a and S3a, fully accounting for the linear scaling with *L*_*y*_ and the periodic oscillations with *L*_*x*_.

The good agreement between the flexible atomistic simulation results and the predictions of the coarse-grained analytical model for rigid interfaces allows us to identify the physical origin of the different features appearing in the force scaling curves. First, when integrating over complete moiré supercells, the contribution of the periodic functions appearing in the potential function of eq 1 vanishes. As such, the main contribution to the sliding energy barrier, and hence to the static friction, is attributed to incomplete moiré tiles near the flake edges. Specifically, the maxima and minima of the static friction force as a function of flake dimensions correspond to in-phase (constructive) and antiphase (destructive) evolution of incomplete moiré tiles at different edges. For aligned rectangular flakes, this effect can be readily demonstrated. On each of the sides that align with the moiré lattice direction (*y*), incomplete edge moiré tiles evolve in-phase during sliding along the *x* direction (see square and rectangular flakes in Supporting Movie S2), leading to constructive contribution to the sliding energy barrier and linear growth of the friction with the *L*_*y*_ side length. On the two sides that are parallel to the sliding direction, the exit of edge moiré tiles during sliding is compensated by the entrance of new moiré tiles on the same side, resulting in no overall growth of the energy barrier with *L*_*x*_. Nonetheless, variation of *L*_*x*_ controls the relative phase of incomplete moiré tile evolution along the two *L*_*y*_ sides, resulting in static friction oscillations of period λ_s_^(*x*)^ ∼ $\sqrt{3}$ λ_m_/2.

We note that the correspondence between the flexible MD simulation results and the rigid model analytical predictions indicates that elasticity effects are of minor importance for static friction. This can be attributed to the relatively small deformation of moiré superstructures in the flexible system with respect to that in the rigid model, including at the rim of the slider. Residual elasticity effects are manifested at the minima of the friction force oscillation with contact dimensions. Here, the rigid model predicts zero friction due to perfect compensation of incomplete moiré tiles at different edges,^39^ whereas the atomistic simulations demonstrate finite friction with nonzero scaling of the lower oscillation envelope. A similar good agreement between MD results and the analytical model predictions is also observed for triangular and hexagonal shaped flakes. (See Supporting Sections 3 and 4).

### Energy Dissipation Analysis

Kinetic friction in our system is also expected to be dominated by edge effects. To demonstrate this, we analyzed the spatial distribution of energy dissipation channels across the flake during sliding dynamics. We find that heat is mainly generated at the corners of the bottom graphene flake layer, where during unpinning local in-plane vibrational modes are excited (see Supporting Figure S7 and Movie S1). This heat propagates via interlayer interactions to the middle graphene and *h*-BN layers, where it is dissipated (see Simulation Setup and Supporting Section 1), allowing us to evaluate the dissipated power (see Supporting Section 5). Notably, energy is found to dissipate mainly via in-plane motion along the sliding direction within the damped Gr layer *l*_2_ (see Supporting Section 5). The power maps presented in Figure 3 demonstrate dominance of corner energy dissipation (see SM Sec. 5.3 for a definition of corner regions in the context of energy dissipation). This is attributed to the higher flexibility of atoms in these regions that allows for forming local lattice commensurability with the adjacent layers. In aligned or marginally twisted heterogeneous interfaces, in addition to rim atom pinning effects,^8,23^ this leads to elastic pinning of incomplete moiré tiles, which dominates friction (see Supporting Movie S2). Notably, we find that the sharper the corner is the higher the energy dissipation power is, such that equilateral hexagonal flakes dissipate less than square and rectangular flakes, which, in turn, dissipate less than equilateral triangular flakes with similar dimensions. In particular, for triangular flakes, this elastic pinning is sufficient to induce local corner stacking instability (ABA ⇌ ABC) during sliding (see Supporting Movie S3), which results in enhanced energy dissipation compared to other polygonal flakes. This behavior is in stark contrast to the case of laterally periodic incommensurate layered interfaces that lack edges and corners (see Supporting Movie S4). The friction in such interfaces is considerably lower and dominated by out-of-plane surface atom motion rather than in-plane elastic edge and corner pinning (see Supporting Section 5).^9^

**Figure 3:**
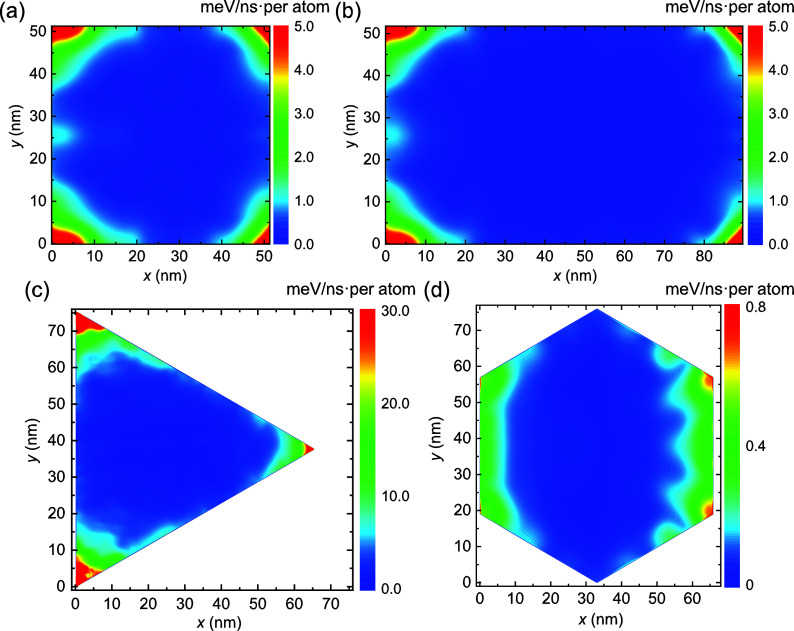
Dissipation power distribution maps for different flake geometries. Energy dissipation maps of the middle layer of (a) square, (b) rectangular (with an aspect ratio of $\sqrt{3}$ ), (c) triangular, and (d) hexagonal graphene flakes measured for system dimensions that correspond to local maxima in the kinetic friction with side length. Energy dissipation power is calculated by averaging over three sliding periods.

The length, *L*_*y*_, of the polygon side that is perpendicular to the sliding direction dictates the pattern evolution of the incomplete moiré tiles at the corners (see yellow arcs in Figure 1c) during sliding, inducing kinetic friction oscillations with a period of λ_k_^(*y*)^ = λ_m_ (see Figures S11 and 2b). Residual energy dissipation appearing mainly along the perpendicular sides (see, e.g., Figure 3d), also associated with elastic pinning of incomplete moiré tiles, is responsible for the linear growth of friction with flake dimensions observed in, e.g., Figure S3b. We note that since the contribution of the inner flake surface and of polygon sides that lie parallel to the sliding direction is minor, square and rectangular shaped flakes demonstrate similar kinetic friction scaling behavior with system dimensions.

The distinct behavior exhibited by the static and kinetic friction with system dimensions (see Figure 2) results from the fact that different mechanisms dominate the two phenomena. While the contribution of incomplete edge moiré tiles dictates static friction,^39^ it is elastic pinning that dominates kinetic friction, where unpinning events lead to significant corner fluctuations associated with enhanced energy dissipation.

### Frictional Scaling Laws

The mechanism revealed above for corner and edge energy dissipation allows us to devise general frictional scaling laws with contact area for layered heterojunctions. Table 1 presents the scaling of the upper envelope of the static friction oscillations for various flake shapes obtained using the analytical rigid sliding model presented above (see eq 3 and Supporting Section 4). As shown in Figure S4a–c, these expressions provide excellent agreement with MD simulation results. Notably, the static friction scaling is dominated by flake side contribution that is proportional to the square root of the contact area. This further supports our conclusion that corner and inner surface area contributions to static friction are of minor importance.

**Table 1:** Analytical Expressions for the Upper Envelopes of the Static Friction Dependence of Interface Area for Various Contact Shapes

shape	upper envelope *F*_s,rigid_^up^ (*A*)
square	$\frac{4}{9}\frac{\lambda_{m}U_{0}}{a_{h\mathrm{BN}}}A^{1/2}$
rectangular	$\frac{4}{9\sqrt[{4}]{3}}\frac{\lambda_{m}U_{0}}{a_{h\mathrm{BN}}}A^{1/2}$
hexagonal	$\frac{\left(\sqrt{3}+\sqrt{11}\right)\sqrt{5\sqrt{3}+\sqrt{11}}}{36}\frac{\lambda_{m}U_{0}}{a_{h\mathrm{BN}}}A^{1/2}$
triangular	$\frac{8}{9\sqrt[{4}]{3}}\frac{\lambda_{m}U_{0}}{a_{h\mathrm{BN}}}A^{1/2}$

For kinetic friction, an analytical treatment, similar to the rigid model for static friction, is lacking. Nonetheless, considering the three main contributions for kinetic friction, namely: (i) corners; (ii) edges; and (iii) internal surface, we can suggest the following phenomenological scaling law

4 $$F_{k}^{\mathrm{up}}\left(A\right)=a_{k}+b_{k}A^{1/2}+c_{k}A$$

where *a*_k_ represents the constant corner contribution, *b*_k_ represents the side contribution per unit length, and *c*_k_ represents the inner surface atomic scale and moiré friction components per unit area.^9,11,36^ Since in the studied nanoscale incommensurate heterogeneous interfaces the corner and edge contributions are expected to dominate over the surface counterpart, the last term in eq 4 can be neglected. The good fit obtained for this expression with the upper and lower envelopes of the kinetic friction dependence on contact area for all flake shapes considered (see thick and thin solid lines, respectively, in Figure 4a–c) supports this proposition. In sharp contrast to the static friction case, the fits to the kinetic friction results yield a dominant contribution of the corner dynamics for the flake dimensions considered (up to 10^4^ nm^2^ in area). The fitted *a*_k_ and *b*_k_ values obtained for the different flake shapes, as appearing in Figure 4a–c, follow an order dictated by the sharpness of the corner angle, consistent with the picture that emerged above from the energy dissipation maps.

**Figure 4:**
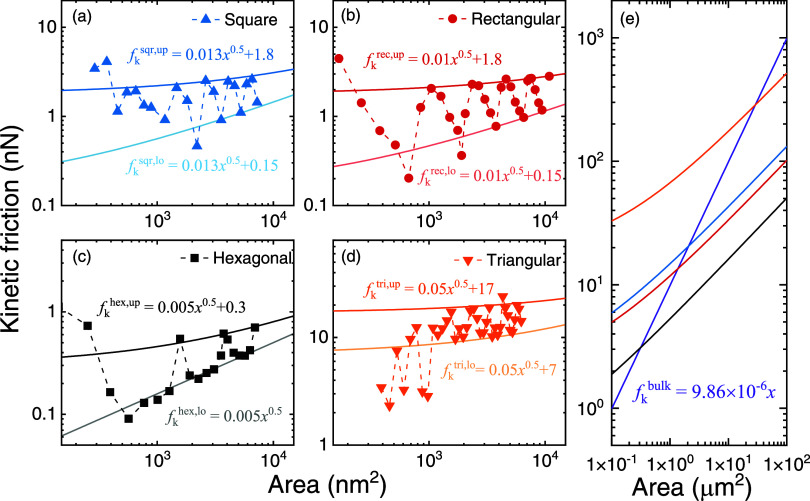
Kinetic friction scaling with contact dimensions. Kinetic friction scaling for (a) square; (b) rectangular; (c) hexagonal; and (d) triangular shaped graphene flakes sliding on an *h*-BN substrate. Solid symbols connected by dashed lines represent atomistic MD simulation results. Dark (light) solid lines are fitted against the upper (lower) envelopes using eq 4 with *c*_k_ = 0. (e) Extrapolation to large contact area demonstrating the crossover between corner, edge, and surface dominated friction (colored according to panels (a–d)). The violet solid line indicates the bulk prediction based on friction values obtained via periodic boundary conditions simulations.

The analyses presented above, allow us to evaluate the regimes at which each friction component dominates. To that end, the upper envelope fits presented in Figures 4 and S4, which assume negligible inner-surface contribution, are used to extrapolate the corner and edge friction components to mesoscale contact areas. This can be compared to the inner-surface contribution by performing separate periodic boundary conditions simulations, that eliminate edge and corner effects and allow us to extract the inner surface contribution to the static (*c*_s_) and kinetic (*c*_k_) friction per unit area (see Supporting Section 3). Figures 4d and S4d present the corresponding extrapolations demonstrating the shape-dependent crossover from corner and edge to surface dominated friction. Specifically, for the kinetic friction, eq 4 can be used to evaluate the transition point between corner to edge dominated friction via the requirement that *a*_k_ ≈ *b*_k_ *A* ^1/2^, yielding a side length of *l* ≈ *a*_k_/*b*_k_ = 340, 139, 180, 60 nm for triangular, square, rectangular, and hexagonal flakes, respectively. Similarly, the transition point to bulk dominated friction is obtained from the requirement that *b*_k_ *A* ^1/2^ = *c*_k_ *A*, yielding a side length of *l* ≈ *b*_k_/*c*_k_ that falls in the range *O*(100 nm)–*O*(10 μm) depending on the shape of the flake. A similar analysis for static friction yields a transition from edge to surface dominated friction at a larger side length of *O*(100 μm). These scaling transition points are larger than those previously predicted for circular flakes,^11^ and are consistent with most experimental observations demonstrating edge dominated friction (manifested as sublinear friction force scaling with contact area) in the *O*(100 nm)–*O*(1 μm) side length range.^18,19,21,22,28,31–34^

### Effect of Interfacial Twist

The results presented above are given for aligned heterogeneous contacts. To evaluate the effect of marginal twisting, which in the case of rigid sliding leads to no static friction scaling with contact dimensions,^39^ we performed sliding simulations of square and triangular flakes, twisted by an angle of θ = 1° with respect to the armchair direction of the underlying *h*-BN surface, and sliding along the same scanline used for the aligned interfaces. In this configuration, the moiré superlattice of period 9.9 nm is rotated by 44.5° with respect to the *h*-BN lattice vectors.

Figure 5 presents the static (panels a and b) and kinetic (panels c and d) friction forces as a function of contact dimensions for square (panels a and c) and triangular (panels b and d) flakes. Both static and kinetic friction forces are significantly lower than those obtained for the aligned heterogeneous interface. Similar to the aligned case, kinetic friction is dominated by corner energy dissipation (see Figure 6) that results from elastic pinning of incomplete moiré tiles (see Suppporting Movie S5). Due to their sharper corner angle, triangular flakes exhibit larger kinetic friction than their square counterparts at the contact dimensions considered. The enhanced kinetic friction observed for the smaller square flakes considered (*L*_s_ < 30 nm in Figure 5c) and all triangular flakes results from sliding induced ABA⇌ABC corner stacking instability, due to strong elastic pinning of incomplete moiré tiles (see Supporting Movie S6). Notably, apart from some periodic variations no overall scaling of static or kinetic friction with contact area is observed up to the largest system considered, which is consistent with the rigid sliding model predictions.^39^

**Figure 5:**
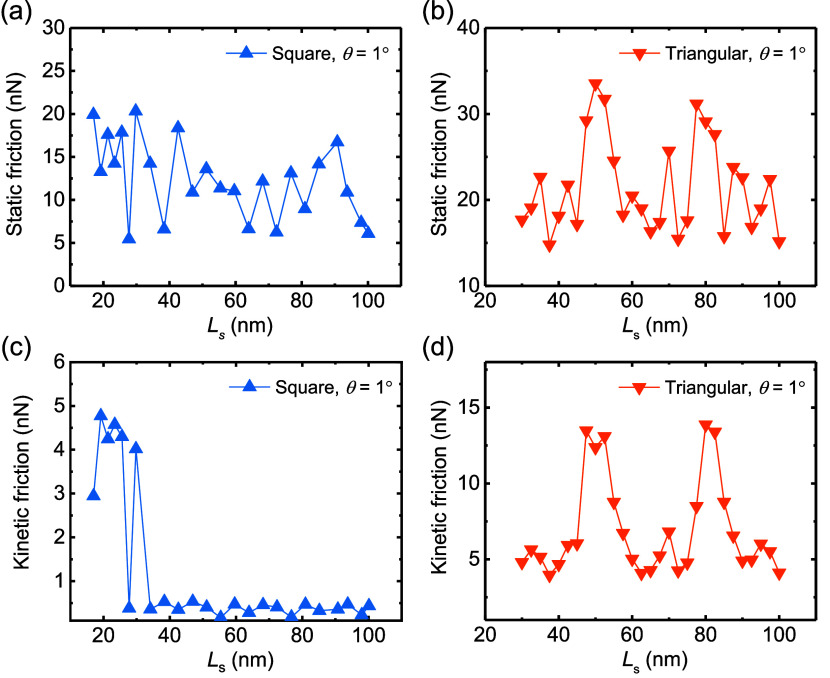
Dependence of the static and kinetic friction forces on the dimensions of marginally twisted (θ = 1°) square and triangular heterogeneous Gr/*h*-BN contacts. Static friction force as a function of contact side length for square (a) and triangular (b) contacts. (c) and (d) are the same as (a) and (b) for the kinetic friction.

**Figure 6:**
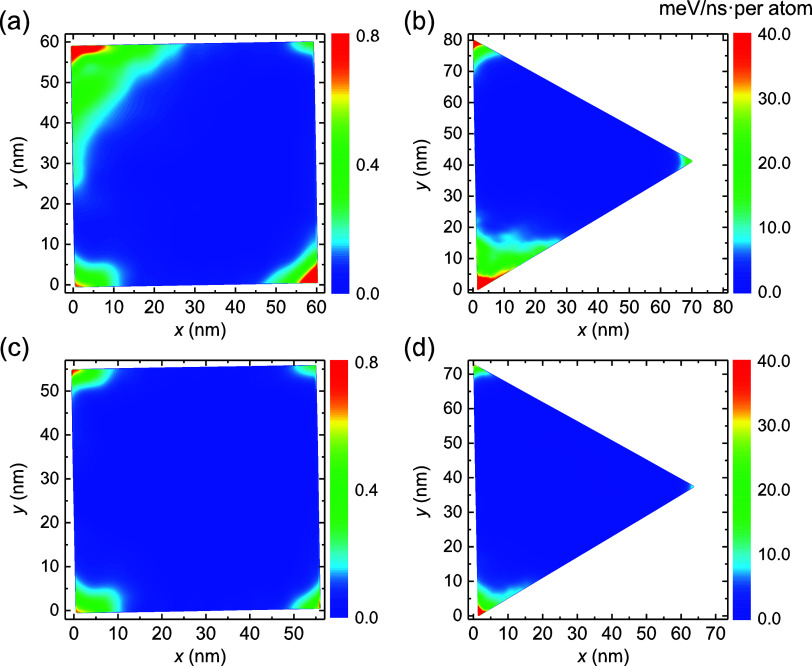
Energy dissipation power distribution maps for marginally twisted (θ = 1°) square and triangular flakes. Energy dissipation maps of the middle layer of (a) square and (b) triangular graphene flakes measured for system dimensions that correspond to local maxima in the kinetic friction with side length. (c) and (d) are the same as (a) and (b), respectively, for local minima in the kinetic friction. Energy dissipation power is calculated by averaging over three sliding periods.

## Conclusions

The results presented above demonstrate that elasticity effects can dominate kinetic friction of incommensurate layered material interfaces already at the nanometer scale. A new energy dissipation mechanism is revealed, involving incomplete moiré tile pinning at the corners and sides of polygonal sliders. The sharpness of the corners and the relative orientation of the sides with respect to the moiré superlattice dictate the magnitude of energy dissipation. While both corner and edge dissipation contribute to the kinetic friction, the static friction is found to be dominated by edge effects. The discovered mechanism is demonstrated for aligned and for marginally twisted heterogeneous graphene/*h*-BN interfaces. The former present linear scaling with side length, consistent with experimental observations,^19,34,36^ whereas the latter do not demonstrate such scaling, thus allowing to achieve large-scale superlubricity. For the aligned contacts, a transition from edge to surface dominated static and kinetic friction is found at *O*(100 μm) and *O*(100 nm–10 μm, depending on the shape of the flake), respectively. We note that preliminary calculations indicate that increasing the thickness of the slider does not modify the underlying energy dissipation mechanism (see Supporitng Sections 3 and 5). Nonetheless, while static friction is insensitive to slider thickness, kinetic friction is found to grow with increasing number of layers, which is attributed to the higher phonon density of states. We therefore offer several control parameters (i.e., corner angle, lateral dimensions, stack thickness, and twist angle) toward the shape tailoring of the frictional properties of heterogeneous finite layered material contacts. While other factors, such as surface roughness, grain boundaries, contaminants, and loading mode may contribute to the frictional behavior of layered contacts,^53–55^ the general design rules devised herein, may promote the development of energy efficient and wear-free interfaces for practical applications in nano- and micro-electro-mechanical systems.

## Methods

All simulations were conducted using the LAMMPS package.^49,50^ The intralayer interactions were described by the REBO^41^ potential for graphene and the Tersoff^42^ potential for *h*-BN. The interlayer interactions were accounted for by the registry-dependent interlayer potential (ILP),^43–46^ with refined parameters.^47,48^ Prior to performing the friction simulations, the system was subjected to structural relaxation using the FIRE algorithm^56,57^ with a force criterion of 10^–3^ eV/Å. During the relaxation process, the lateral position of the topmost rigid graphene layer was kept fixed, whereas its vertical position was free to adjust. After structural minimization, sliding simulations were performed under zero normal load and at zero temperature, aiming to highlight the underlying energy dissipation mechanism by avoiding thermal noise. To remove the heat generated during shear, viscous damping was applied to the velocities (relative to the center of mass of the layer) of the atoms in the second Gr layer (*l*_2_) and the second *h*-BN layer (*l*_5_) with a damping coefficient η = 1.0 ps^–1^ in all three directions. This allows heat to dissipate via both the slider and the substrate aiming to mimic experimental scenarios, while introducing minor disturbance to the dynamics of atoms at the shear interface.

## Supplementary Material















## Notes

Simulation protocol; lateral force traces and friction force calculation; additional friction simulation results; analytical model for static friction; frictional dissipation analysis (PDF)
Sliding simulation of a square flake with zoom-in views of the corners (Movie S1) (MP4)
Moiré evolution in the bottom flake layer for different geometries in the aligned configuration (Movie S2) (MP4)
Stacking instability in the corner regions of a triangular flake in the aligned configuration (Movie S3) (MP4)
Moiré evolution in the bottom layer of a perfect periodic flake (Movie S4) (MP4)
Moiré evolution in the bottom layer of 1° twisted square and triangular flakes (Movie S5) (MP4)
Stacking instability in the corner region of a 1° twisted triangular flake (Movie S6) (MP4)

## References

[1] Hod O., Meyer E., Zheng Q., Urbakh M. (2018). Structural superlubricity
and ultralow friction across the length scales. Nature.

[2] Dienwiebel M., Verhoeven G. S., Pradeep N., Frenken J. W. M., Heimberg J. A., Zandbergen H. W. (2004). Superlubricity of Graphite. Phys.
Rev. Lett..

[3] Liu Z., Yang J., Grey F., Liu J. Z., Liu Y., Wang Y., Yang Y., Cheng Y., Zheng Q. (2012). Observation
of microscale superlubricity in graphite. Phys.
Rev. Lett..

[4] Filippov A. E., Dienwiebel M., Frenken J. W. M., Klafter J., Urbakh M. (2008). Torque and
Twist against Superlubricity. Phys. Rev. Lett..

[5] Leven I., Krepel D., Shemesh O., Hod O. (2013). Robust Superlubricity
in Graphene/*h*-BN Heterojunctions. J. Phys. Chem. Lett..

[6] Mandelli D., Leven I., Hod O., Urbakh M. (2017). Sliding friction of
graphene/hexagonal–boron nitride heterojunctions: a route to
robust superlubricity. Sci. Rep..

[7] Song Y., Mandelli D., Hod O., Urbakh M., Ma M., Zheng Q. (2018). Robust microscale superlubricity
in graphite/hexagonal boron nitride
layered heterojunctions. Nat. Mater..

[8] Liao M., Nicolini P., Du L., Yuan J., Wang S., Yu H., Tang J., Cheng P., Watanabe K., Taniguchi T. (2022). UItra-low friction and edge-pinning effect in large-lattice-mismatch
van der Waals heterostructures. Nat. Mater..

[9] Mandelli D., Ouyang W., Hod O., Urbakh M. (2019). Negative Friction Coefficients
in Superlubric Graphite-Hexagonal Boron Nitride Heterojunctions. Phys. Rev. Lett..

[10] Wang J., Khosravi A., Vanossi A., Tosatti E. (2024). Colloquium: Sliding
and pinning in structurally lubric 2D material interfaces. Rev. Mod. Phys..

[11] Wang J., Cao W., Song Y., Qu C., Zheng Q., Ma M. (2019). Generalized
Scaling Law of Structural Superlubricity. Nano
Lett..

[12] Wang J., Ma M., Tosatti E. (2023). Kinetic friction of
structurally superlubric 2D material
interfaces. J. Mech. Phys. Solids.

[13] Sharp T. A., Pastewka L., Robbins M. O. (2016). Elasticity
limits structural superlubricity
in large contacts. Phys. Rev. B.

[14] Müser M. H. (2004). Structural
lubricity: Role of dimension and symmetry. Europhys.
Lett..

[15] Gao X., Ouyang W., Hod O., Urbakh M. (2021). Mechanisms of frictional
energy dissipation at graphene grain boundaries. Phys. Rev. B.

[16] Gao X., Ouyang W., Urbakh M., Hod O. (2021). Superlubric polycrystalline
graphene interfaces. Nat. Commun..

[17] Gao X., Urbakh M., Hod O. (2022). Stick-Slip
Dynamics of Moiré
Superstructures in Polycrystalline 2D Material Interfaces. Phys. Rev. Lett..

[18] Hartmuth F., Dietzel D., de Wijn A. S., Schirmeisen A. (2019). Friction vs.
Area Scaling of Superlubric NaCl-Particles on Graphite. Lubricants.

[19] Barabas A. Z., Sequeira I., Yang Y., Barajas-Aguilar A. H., Taniguchi T., Watanabe K., Sanchez-Yamagishi J. D. (2023). Mechanically
reconfigurable van der Waals devices via low-friction gold sliding. Sci. Adv..

[20] Song Y., Gao X., Pawlak R., Huang S., Hinaut A., Glatzel T., Hod O., Urbakh M., Meyer E. (2024). Non-Amontons frictional behaviors
of grain boundaries at layered material interfaces. Nat. Commun..

[21] Dietzel D., Brndiar J., Štich I., Schirmeisen A. (2017). Limitations
of Structural Superlubricity: Chemical Bonds versus Contact Size. ACS Nano.

[22] Qu C., Wang K., Wang J., Gongyang Y., Carpick R. W., Urbakh M., Zheng Q. (2020). Origin of
Friction in Superlubric
Graphite Contacts. Phys. Rev. Lett..

[23] Liu Y., Ren J., Kong D., Shan G., Dou K. (2023). Edge-pinning effect
of graphene nanoflakes sliding atop graphene. Mater. Today Phys..

[24] Ying P., Natan A., Hod O., Urbakh M. (2024). Effect of
Interlayer
Bonding on Superlubric Sliding of Graphene Contacts: A Machine-Learning
Potential Study. ACS Nano.

[25] Ouyang W., Cheng Y., Ma M., Urbakh M. (2020). Load-velocity-temperature
relationship in frictional response of microscopic contacts. J. Mech. Phys. Solids.

[26] Wang K., He Y., Cao W., Wang J., Qu C., Chai M., Liu Y., Zheng Q., Ma M. (2022). Structural
superlubricity with a
contaminant-rich interface. J. Mech. Phys. Solids.

[27] Müser M. H., Wenning L., Robbins M. O. (2001). Simple
Microscopic Theory of Amontons’s
Laws for Static Friction. Phys. Rev. Lett..

[28] Dietzel D., Ritter C., Mönninghoff T., Fuchs H., Schirmeisen A., Schwarz U. D. (2008). Frictional Duality Observed during Nanoparticle Sliding. Phys. Rev. Lett..

[29] Ouyang W., de Wijn A. S., Urbakh M. (2018). Atomic-scale
sliding friction on
a contaminated surface. Nanoscale.

[30] Deng H., Ma M., Song Y., He Q., Zheng Q. (2018). Structural superlubricity
in graphite flakes assembled under ambient conditions. Nanoscale.

[31] Özoğul A., İpek S., Durgun E., Baykara M. Z. (2017). Structural superlubricity
of platinum on graphite under ambient conditions: The effects of chemistry
and geometry. Appl. Phys. Lett..

[32] Cihan E., İpek S., Durgun E., Baykara M. Z. (2016). Structural lubricity
under ambient conditions. Nat. Commun..

[33] Dietzel D., Feldmann M., Schwarz U. D., Fuchs H., Schirmeisen A. (2013). Scaling Laws
of Structural Lubricity. Phys. Rev. Lett..

[34] Koren E., Lörtscher E., Rawlings C., Knoll A. W., Duerig U. (2015). Adhesion and
friction in mesoscopic graphite contacts. Science.

[35] Li H., Wang J., Gao S., Chen Q., Peng L., Liu K., Wei X. (2017). Superlubricity
between MoS_2_ Monolayers. Adv. Mater..

[36] Li Y., He W., He Q.-C., Wang W. (2024). Contributions of Edge and Internal
Atoms to the Friction of Two-Dimensional Heterojunctions. Phys. Rev. Lett..

[37] Koren E., Duerig U. (2016). Moiré scaling
of the sliding force in twisted
bilayer graphene. Phys. Rev. B.

[38] de
Wijn A. S. (2012). (In)­commensurability, scaling, and multiplicity of
friction in nanocrystals and application to gold nanocrystals on graphite. Phys. Rev. B.

[39] Yan W., Gao X., Ouyang W., Liu Z., Hod O., Urbakh M. (2024). Shape-dependent
friction scaling laws in twisted layered material interfaces. J. Mech. Phys. Solids.

[40] Yan W., Ouyang W., Liu Z. (2023). Origin of frictional scaling law
in circular twist layered interfaces: Simulations and theory. J. Mech. Phys. Solids.

[41] Brenner D. W., Shenderova O. A., Harrison J. A., Stuart S. J., Ni B., Sinnott S. B. (2002). A second-generation reactive empirical bond order (REBO)
potential energy expression for hydrocarbons. J. Phys.:Condens. Matter.

[42] Tersoff J. (1988). New empirical
approach for the structure and energy of covalent systems. Phys. Rev. B.

[43] Kolmogorov A. N., Crespi V. H. (2005). Registry-dependent interlayer potential
for graphitic
systems. Phys. Rev. B.

[44] Leven I., Maaravi T., Azuri I., Kronik L., Hod O. (2016). Interlayer
Potential for Graphene/*h*-BN Heterostructures. J. Chem. Theory Comput..

[45] Leven I., Azuri I., Kronik L., Hod O. (2014). Inter-Layer
Potential
for Hexagonal Boron Nitride. J. Chem. Phys..

[46] Maaravi T., Leven I., Azuri I., Kronik L., Hod O. (2017). Interlayer
Potential for Homogeneous Graphene and Hexagonal Boron Nitride Systems:
Reparametrization for Many-Body Dispersion Effects. J. Phys. Chem. C.

[47] Ouyang W., Mandelli D., Urbakh M., Hod O. (2018). Nanoserpents:
Graphene
Nanoribbon Motion on Two-Dimensional Hexagonal Materials. Nano Lett..

[48] Ouyang W. G., Azuri I., Mandelli D., Tkatchenko A., Kronik L., Urbakh M., Hod O. (2020). Mechanical and Tribological
Properties of Layered Materials under High Pressure: Assessing the
Importance of Many-Body Dispersion Effects. J. Chem. Theory Comput..

[49] Plimpton S. (1995). Fast Parallel
Algorithms for Short-Range Molecular Dynamics. J. Comput. Phys..

[50] Thompson A. P., Aktulga H. M., Berger R., Bolintineanu D. S., Brown W. M., Crozier P. S., in ’t Veld P. J., Kohlmeyer A., Moore S. G., Nguyen T. D. (2022). LAMMPS
- a flexible simulation tool for particle-based materials modeling
at the atomic, meso, and continuum scales. Comput.
Phys. Commun..

[51] Vanossi A., Manini N., Tosatti E. (2012). Static and dynamic
friction in sliding
colloidal monolayers. Proc. Natl. Acad. Sci.
U.S.A..

[52] Ouyang W., Sofer R., Gao X., Hermann J., Tkatchenko A., Kronik L., Urbakh M., Hod O. (2021). Anisotropic Interlayer
Force Field for Transition Metal Dichalcogenides: The Case of Molybdenum
Disulfide. J. Chem. Theory Comput..

[53] Feng S., Xu Z. (2021). Pattern Development and Control of Strained Solitons in Graphene
Bilayers. Nano Lett..

[54] Zhang Y., Li J., Wang Y., Nie J., Wang C., Tian K., Ma M. (2024). Loading Mode-Induced
Enhancement in Friction for Microscale Graphite/Hexagonal
Boron Nitride Heterojunction. ACS Appl. Mater.
Interfaces.

[55] Ying P., Gao X., Berman D., Hod O., Urbakh M. (2025). Scaling-Up of Structural
Superlubricity: Challenges and Opportunities. Adv. Funct. Mater..

[56] Bitzek E., Koskinen P., Gähler F., Moseler M., Gumbsch P. (2006). Structural
Relaxation Made Simple. Phys. Rev. Lett..

[57] Guénolé J., Nöhring W. G., Vaid A., Houllé F., Xie Z., Prakash A., Bitzek E. (2020). Assessment and optimization of the
fast inertial relaxation engine (fire) for energy minimization in
atomistic simulations and its implementation in lammps. Comput. Mater. Sci..

