# Supplementary material for: Frictional Dissipation and Scaling Laws at van der Waals Interfaces: The Role of Edge and Corner Elastic Moiré Pinning
Source: ACS Nano. 2025 Aug 11;19(32):29255–64. doi: 10.1021/acsnano.5c04617 (PMC12369003; doi:10.1021/acsnano.5c04617)
Supplement: Supplementary file 1 [file nn5c04617_si_001.pdf]

Supplementary Material for

"Frictional Dissipation and Scaling Laws at van der Waals Interfaces: The Role of Edge and Corner Elastic Moiré Pinning"

Xiang Gao,[1] Weidong Yan,[2] Wengen Ouyang,[2,3] Ze Liu,[2] Michael Urbakh,[4]*, Oded Hod[4]

[1]CAS Key Laboratory of Mechanical Behavior and Design of Materials, Department of Modern Mechanics, University of Science and Technology of China, Hefei, 230027, Anhui, China

[2]Department of Engineering Mechanics, School of Civil Engineering, Wuhan University, Wuhan, Hubei 430072, China

[3]State Key Laboratory of Water Resources Engineering and Management, Wuhan University, Wuhan, Hubei, 430072, China

[4]Department of Physical Chemistry, School of Chemistry, The Raymond and Beverly Sackler Faculty of Exact Sciences and The Sackler Center for Computational Molecular and Materials Science, Tel Aviv University, Tel Aviv 6997801, Israel

*Corresponding author. Email: urbakh@tauex.tau.ac.il

In this supplemental material, we provide additional details on the following subjects:

1. Simulation Protocol
2. Lateral Force Traces and Friction Force Calculation
3. Additional Friction Simulation Results
4. Analytical Model for Static Friction
5. Frictional Dissipation Analysis

1. Simulation Details

1.1. Simulation Protocol

The simulation protocol used in this work follows that employed in our previous studies of friction over polycrystalline layered material interfaces.[1-3] The graphene/h-BN heterojunction model systems were composed of a trilayer h-BN substrate and a trilayer graphene slider. To mimic a rigidly moving stage, the topmost graphene layer was kept rigid and the bottommost h-BN layer was rigidly fixed at its initial position throughout the simulations. All the rest four intermediate layers were set to be flexible. The intralayer interactions were modeled with the conventional second-generation reactive empirical bond order (REBO) potential[4] and Tersoff potential[5] for graphene and h-BN, respectively, which have been previously demonstrated to be suitable for this task.[6-8] To match the experimental value of the intrinsic lattice mismatch, i.e., 1.8%, between graphene and h-BN, the equilibrium B-N bond length in the Tersoff potential was increased by 0.26% to $d_{\mathrm{BN}} = 1.445957$ Å (compared to the original value of 1.44214 Å), and the C-C bond length was kept at its original value of $d_{\mathrm{cc}} = 1.42039$ Å. This has a negligible effect on the calculated elastic properties.[9] The anisotropic interlayer interactions were described by the registry-dependent interlayer potential (ILP),[10-14] parameterized against density functional theory (DFT) reference calculations using the HSE06 screened-exchange density functional approximation[15] including many-body dispersion (MBD) corrections,[16] which provides a reliable description of van der Waals interactions in layered materials in both the equilibrium and sub-equilibrium regimes.[14, 17]

During the sliding simulations, the center-of-mass of the top layer was pulled with a constant velocity of v_0 = 5 m/s in the armchair (x) direction of the h-BN lattice. Frictional heat dissipation in the moving stage and the substrate, was modeled by applying viscous damping with a damping coefficient of $\eta = 1.0$ ps^{-1} to the atoms in the second graphene flake layer (l_2) and the second h-BN substrate layer (l_5) in all three directions (see main text Fig. 1). The corresponding damping force terms are given by:

$$\mathbf{f}_{\mathrm{damp}}^{i,l_2}(t) = -m_{\mathrm{C}}\eta\left[v_x^{i,l_2}(t) - v_0\right]\hat{\mathbf{x}} - m_{\mathrm{C}}\sum_{\alpha=y,z}\eta\, v_\alpha^{i,l_2}(t)\hat{\boldsymbol{\alpha}},$$

$$\mathbf{f}_{\mathrm{damp}}^{i,l_5}(t) = -m^i\sum_{\alpha=x,y,z}\eta v_\alpha^{i,l_5}(t)\,\hat{\boldsymbol{\alpha}},\ m^i = m_{\mathrm{B}} \text{ or } m_{\mathrm{N}}, \tag{S1}$$

where $m_{\mathrm{C}}, m_{\mathrm{B}}$, and m_{N} are the atomic masses of carbon, boron, and nitrogen atoms, respectively, $v_\alpha^{i,l_k}(t)$ is the α Cartesian velocity component of the ith atom in layer l_k ($k = 2$ or 5) at time t, and $\hat{\boldsymbol{\alpha}} = \hat{\mathbf{x}}, \hat{\mathbf{y}}, \hat{\mathbf{z}}$ are the unit vectors in the x, y and z directions, respectively. Note that the damping in the sliding direction of layer l_2 is proportional to the relative atomic velocities, with respect to the driving velocity, v_0, i.e. to the internal degree-of-freedom of the flake within its rest reference frame.

We note that our choice of viscous damping terms is well justified as long as the results do not strongly depend on the chosen damping rate (see Ref. 1 and Sec. 3.3). This has been previously demonstrated for the case of stick-slip sliding (also considered herein, see Fig. S6), where the dynamics is dominated by barrier crossing processes.[1] Further support for using isotropic damping rates was provided by the fact that our computational predictions for graphene grain boundaries based on this choice have been experimentally confirmed.[20]

All simulations were performed using the LAMMPS package.[21, 22] Typically, the sliding simulations last for 3-4 sliding periods to provide sufficient long steady state lateral force traces for the calculation of friction. The calculation of lateral friction forces and their convergence are discussed in detail in Sec. 2.

1.2. Effect of sliding velocity

The simulations presented in the main text have been performed with a sliding velocity of 5 m/s, which is orders of magnitude larger than the velocities studied under typical experimental conditions. This is characteristic of all atomistic sliding simulations in the field of nanotribology and is a consequence of the computational burden involved with evaluating the forces and propagating the equations of motion.

To verify that our main findings are relevant to realistic experimental scenarios, we have repeated some of our simulations both at a lower sliding velocity of $2\ \mathrm{m/s}$ and under quasi-static conditions, where after each propagation step the system is allowed to fully relax (to that end we use the FIRE algorithm[18, 19] with a force convergence criterion of 2×10^{-4} eV/Å) before the next step is executed. The latter, which is highly computationally demanding, represents the low-velocity limit relevant to experiments.

Figure S1 demonstrates that the sliding dynamic at these two extremes is very similar, apart from some post-slip oscillations that are absent in the quasi-static case. The calculated average kinetic friction differs by a factor of ~3 between the $5\ \mathrm{m/s}$ sliding and the quasistatic condition. Hence, though the sliding velocity in our simulations is orders of magnitude higher as compared to experiments, the qualitative nature of our scaling predictions remain valid and relevant and the quantitative differences in the frictional forces are expected to be within a small multiplicative factor.

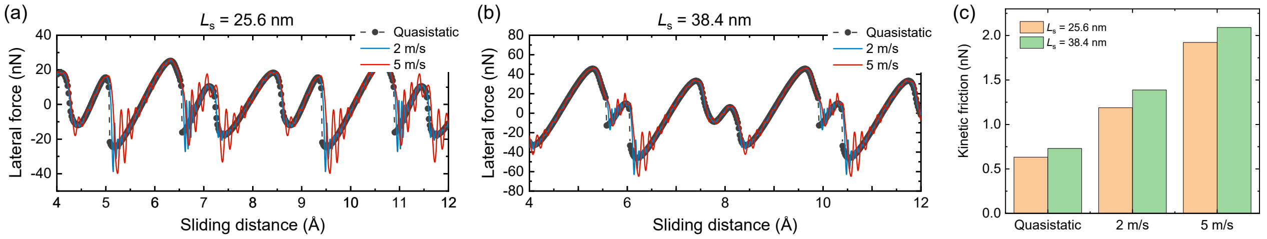

Figure S1. Effect of sliding velocity on the frictional dynamics. Lateral force traces of (a) $25.6 \times 25.6\ \mathrm{nm}^2$ and (b) $38.4 \times 38.4\ \mathrm{nm}^2$ square graphene flakes sliding on *h*-BN at the aligned configuration, calculated under quasistatic conditions (black circles), and for sliding velocities of 2 (blue line) and 5 (red line) $\mathrm{m/s}$. (c) The average kinetic friction forces calculated from the force traces in panels (a, orange) and (b, green).

2. Lateral Force Traces and Friction Force Calculation

The static and kinetic friction forces were estimated from the total lateral force acting on the rigidly driven top graphene layer in the sliding direction. Figure S2(a)-(c) presents typical lateral force traces for square flakes. The lateral force traces exhibit clear periodic patterns with a period of $t_0 = \frac{a_{hBN}^{\text{armchair}}}{v_0} = \frac{4.33787\ \text{Å}}{5\ \text{m/s}} \approx 86.76\ \text{ps}$, corresponding to sliding over one armchair period of h-BN. The static friction, F_s, is defined as the maximum (negative) lateral force during each sliding period. The kinetic friction, F_k, is evaluated by block-average of the (negative) force traces in each sliding period. In Fig. S2(d)-(i), we show the calculated results of F_s and F_k during the first four sliding periods. After some initial transient dynamics during the first sliding period, steady-state is obtained and the static and kinetic friction forces extracted from each period stabilize. Hence, we omit the result of the first period and average over the results of the rest 2-4 periods to evaluate the static and kinetic friction forces.

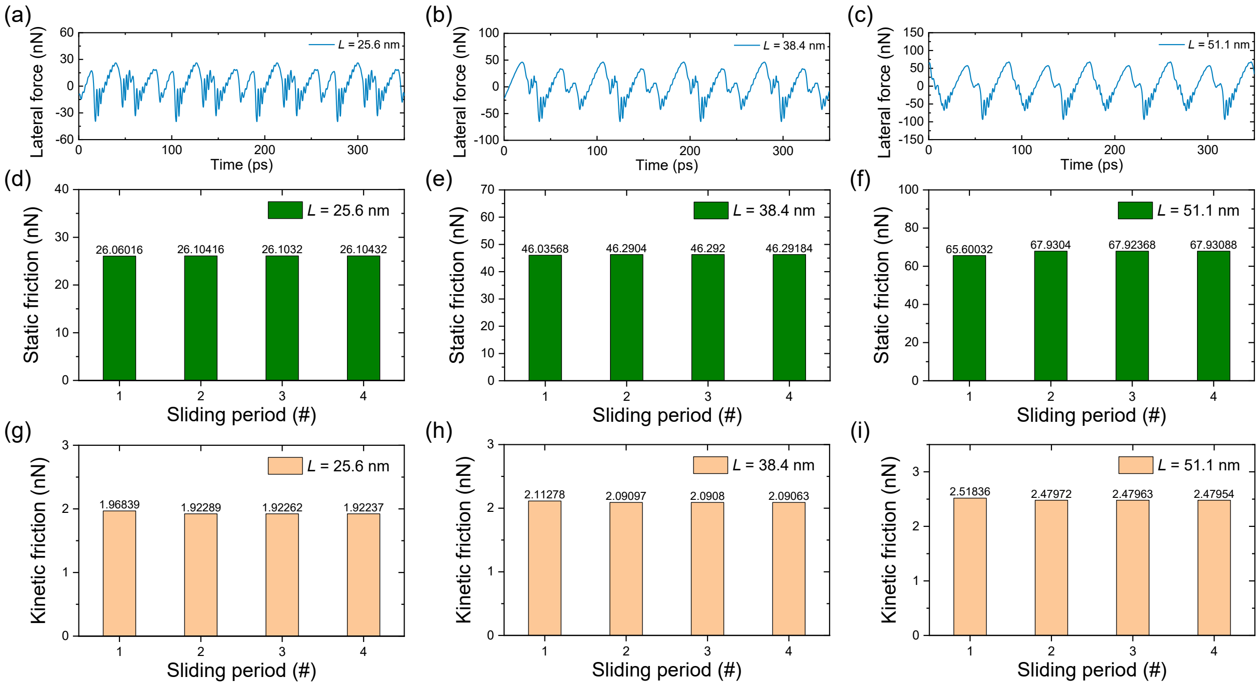

Figure S2. Friction force calculation. (a)-(c) Representative negative lateral force traces acting on the top rigid graphene layer in the sliding direction for square flakes of three different sizes calculated at zero temperature and under zero external normal load. (d)-(f) The corresponding static friction calculated in each sliding period. (g)-(i) Same as panels (d)-(f) but for kinetic friction.

3. Additional Friction Simulation Results

3.1. Size dependence of friction forces for triangular and hexagonal flakes

In Fig. 2 of the main text, we presented results for the contact size dependence of friction for rectangular flakes with different aspect ratio. For completeness, we provide here similar results for the static and kinetic friction forces of square flakes, rectangular flakes with fixed aspect ratio, and equilateral triangular and hexagonal flakes. Fig. S3(a) demonstrates that for square flakes and rectangular flakes of fixed aspect ratio the static friction force behavior with L_x match. Similarly, Fig. S3(b) demonstrates good correspondence between the kinetic friction force dependence on L_y of the two systems. As shown in Fig. S3(c)-(d), hexagonal flakes exhibit periods of $\lambda_\mathrm{m}/2$ and λ_m for static and kinetic friction, respectively. In contrast, for triangular flakes, both static and kinetic friction demonstrate a period of λ_m (Fig. S3(e)-(f)). The periodicities and growth of the static friction upper envelopes for both shapes can be well described by the analytical models in Sec. 4. The lower envelopes of the hexagonal flakes grow with contact size, deviating from the predictions of the rigid analytical model. All these behaviors are similar to this observed for rectangular and square flakes as shown in Fig. 2a of the main text.

We note that the different periodicities for the static friction between hexagonal and triangular flakes are attributed to the different side length required for the mutual compensation of incomplete moiré tiles at different edges. For instance, considering the two parallel sides along the y direction, full compensation occurs with an inter-side separation period of $\sqrt{3}\,\lambda_\mathrm{m}/2$, which corresponds to a hexagon side-length of $\lambda_\mathrm{m}/2$. For triangular shape, complete compensation cannot be achieved, due to the absence of parallel sides, thus only partial compensation is observed with a static friction period of λ_m.

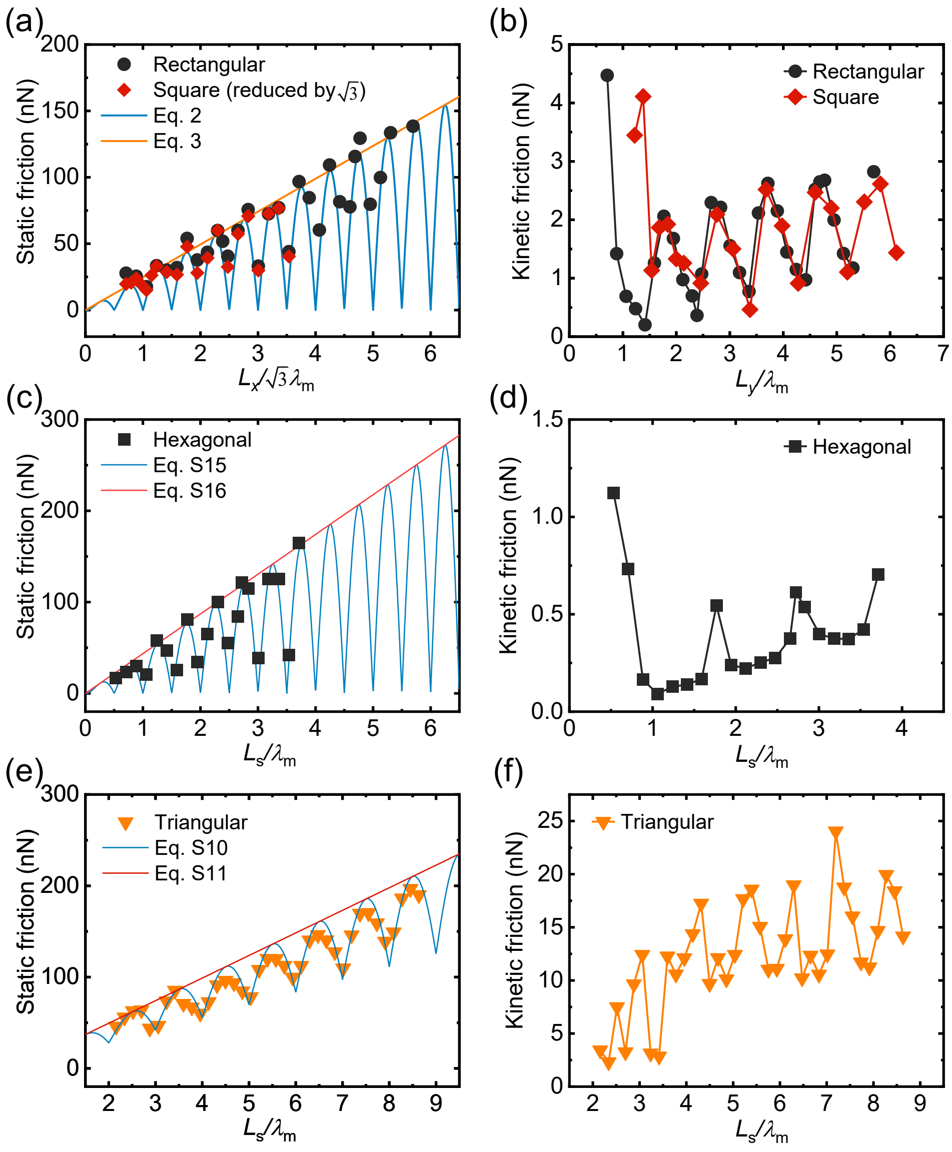

Figure S3. Size dependence of (a), (c), (e) static and (b), (d), (f) kinetic friction for (a)-(b) square and rectangular, (c)-(d) hexagonal and (e)-(f) triangular shaped flakes. The rectangular flakes considered have a fixed aspect ratio of $L_x/L_y = \sqrt{3}$. In panel (a), to account for the difference in contact dimensions, the values of F_s for square flakes are divided by a factor of $\sqrt{3}$. The solid lines in panels (a) are theoretical predictions obtained using Eqs. (2) and (3) in the main text.

Figure S4(a)-(d) presents the scaling behavior of the static friction of polygonal flakes with contact area. It is seen that the upper envelopes for each shape are well described by the analytical models presented in Table 1 of the main text, and the lower envelopes fit well the same scaling law of $F_s \propto A^{\frac{1}{2}}$. These suggested that the static friction is dominated by the edge effects, in contrast to kinetic friction, which is dominated by corner effects up to hundreds of nanometers in dimension. The

transition from edge dominated to area dominated static friction is estimated to be on the order of hundred micrometers (see Fig. S4(e)).

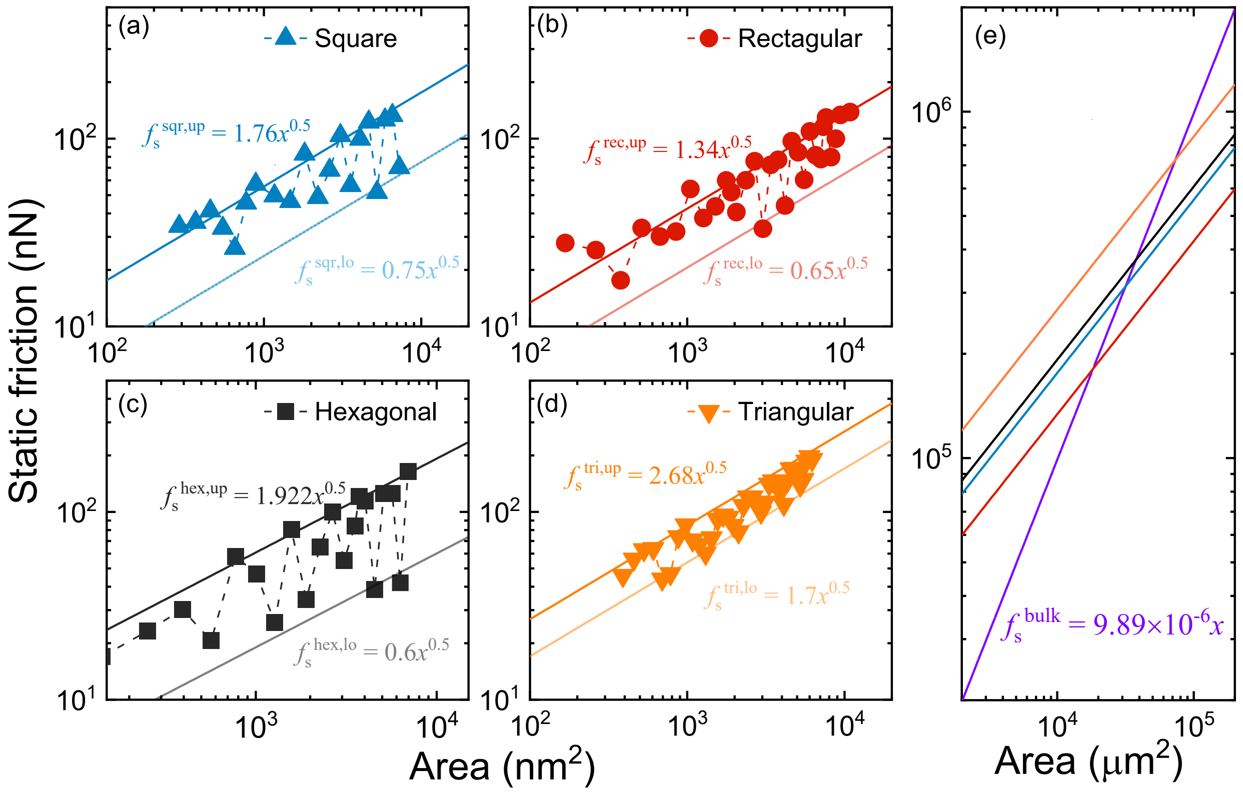

Figure S4. Static friction scaling with contact dimensions. (a)-(d) Static friction scaling for (a) square; (b) rectangular; (c) hexagonal; and (d) triangular shaped graphene flakes sliding on an *h*-BN substrate. Solid symbols connected by dashed lines represent MD simulation results. Dark solid lines (colored according to the corresponding symbol) are calculated for the upper envelopes using the expressions appearing in Table 1 of the main text with $U_0 = 4.5$ meV/Å^2. Light solid lines are fits of the lower envelopes to a scaling law proportional to the side length, $F_s \propto A^{\frac{1}{2}}$. (e) Extrapolation to large contact area demonstrating the cross-over between edge and surface dominated friction (colored according to panels (a)-(d)). The violet solid line indicates the bulk prediction based on friction values obtained via periodic boundary conditions simulations.

3.2. Friction contribution of inner surface regions

To evaluate the frictional contribution of inner surface regions, we performed sliding simulations with laterally periodic model systems, where the effects of edges and corners are excluded. To this end, we built laterally periodic graphene/*h*-BN heterojunctions in the aligned configuration with 112×112 graphene and 110×110 *h*-BN unit cells (lateral dimension 47.73×27.55 nm^2), which gives a lattice

mismatch of 1.82%, close to the experimental value of ~1.8% used for the finite flake system simulations. Accordingly, to fulfill the periodic boundary condition, we kept the equilibrium lattice constant value of $d_{\mathrm{cc}} = 1.42039$ Å for graphene, as given by the REBO potential, while shifted the equilibrium lattice constant value for h-BN by an increment of 0.004072573 Å from the original Tersoff potential value to $d_{\mathrm{BN}} = 1.446215273$ Å. This leads to a slightly smaller moiré superstructure period of $\lambda_{\mathrm{m}} \sim 13.78$ nm compared to that in the finite flake systems (~13.91 nm), which is expected to have a minor effect on friction and energy dissipation characteristics.

Figure S5 presents the lateral force trace for the periodic supercells of contact area $S \approx 1315.0$ nm^2 at steady state that gives an average friction force of 12.97 pN, from which we can extract the surface kinetic friction density coefficient of Eq. 4 in the main text, $c_{\mathrm{k}} = 9.86 \times 10^3$ N/m^2. Similarly, from the maximum of the force trace, we obtain a surface static friction density coefficient of $c_{\mathrm{s}} = 9.89 \times 10^3$ N/m^2.

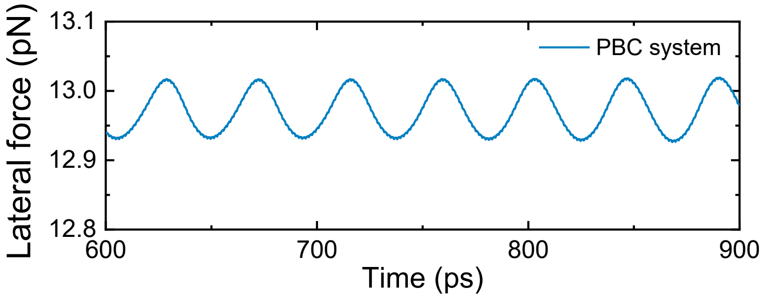

Figure S5. Steady-state lateral force trace for laterally periodic aligned graphene/h-BN interface composed of 112×112 graphene and 110×110 h-BN unit cells.

3.3. Effect of slider thickness on frictional scaling

Similar to serially connected springs, the lateral stiffness of the flake slider depends inversely its thickness, which thus may have an impact on the energy dissipation and frictional behaviors. To evaluate the effect of thickness, we conducted sliding simulations with thicker square sliders of 6 layers and 20 layers, where viscous damping is applied to all flexible flake layers except the interfacial ones. As shown in Fig. S6(a)-(b), the static friction for thicker sliders exhibits similar periodicity and growth rate with contact dimensions, following the analytical model (Eq. 3 in the main text). For kinetic friction, it is seen that the fluctuation magnitude grows with the increase of thickness with a slight shift in the maxima positions. Along with the energy dissipation power distributions that demonstrate edge and corner dominated friction for the 6-layered flakes, as well (Fig. S12), we conclude that the qualitative nature of our predictions is independent of flake thickness.

To demonstrate the robustness of our artificial remote damping scheme and its negligible effect on the frictional behavior of the sliding interface, we present in Fig. S6(c)-(d) sliding friction results of the 6-layer square flake system, where viscous damping is excluded from the second flake layer adjacent to the bottom layer of the sliding flake and applied to the remaining three flexible layers above it. The results (labeled as damping_3L) are in excellent agreement with results obtained when all four flexible flake layers above the interfacial one are damped (labeled as damping_4L in the figure).

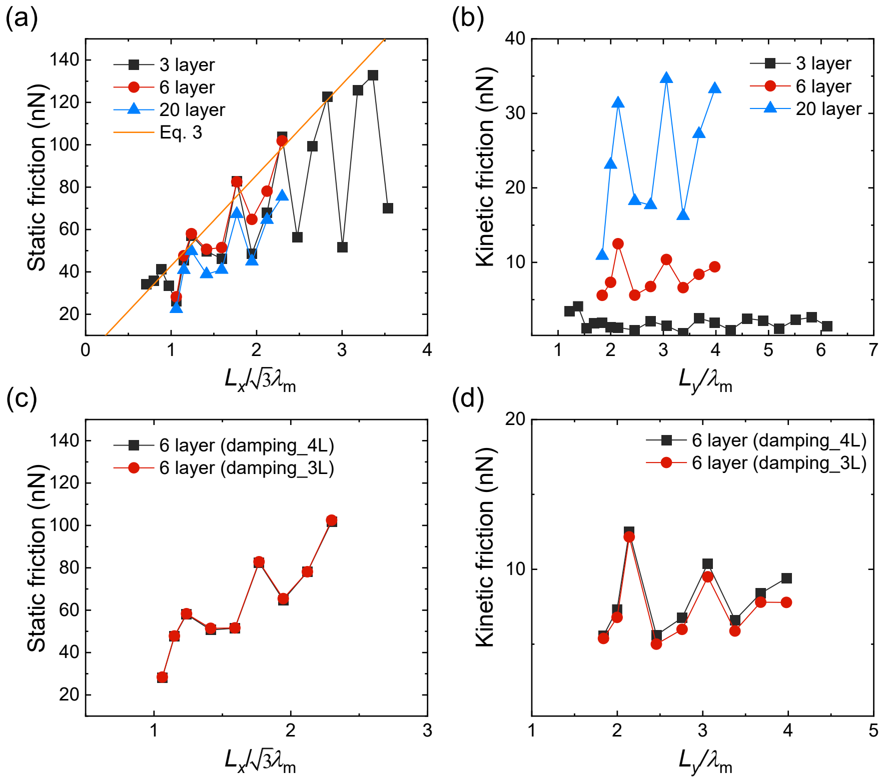

Figure S6. Effect of slider thickness on (a) static friction and (b) kinetic friction for square flake systems. (c)-(d) Demonstration of the robustness of the damping schemes. Comparison of the performance of two damping schemes: (i) damping 3L, where damping is excluded from the layer adjacent to the interfacial graphene layer and (ii) damping 4L, where damping is applied to all flexible graphene flake layers above the interfacial one, for (c) static and (d) kinetic friction of the 6-layer square flake system.

4. Analytical Model for Static Friction

In this section, we provide a detailed derivation for the expressions of static friction for different flake shapes, using the approach presented in Ref. 23. For a rigid graphene/h-BN interface, the interlayer interactions can be approximated by a continuum potential energy density function of the form:[23]

$$U(x,y) = -\frac{2}{9}U_0\left(\frac{3}{2} + 2\cos\frac{2\pi x}{\sqrt{3}\lambda_{\mathrm{m}}}\cos\frac{2\pi y}{\lambda_{\mathrm{m}}} + \cos\frac{4\pi x}{\sqrt{3}\lambda_{\mathrm{m}}}\right), \quad \text{(S2)}$$

where the constant $\frac{3}{2}$ in the parathesis on the right-hand side does not affect the sliding energy corrugation for a slider of given size and shape, thus is omitted in what follows. As described in the main text, the total interlayer energy of a flake of surface area A, whose geometric center is positioned at (x_0, y_0) with respect to a reference frame located at a domain center of the moiré superlattice, can be calculated as $E(A; x_0, y_0) = \iint_S U(x,y)dxdy$, where the integration is performed over the entire flake surface. The corresponding static friction can be evaluated as the maximal derivative of the interlayer energy along the scanline (chosen here to be parallel to the x axis): $F_{\mathrm{s,rigid}} = \left(\frac{\lambda_{\mathrm{m}}}{a_{h\mathrm{BN}}}\right)\max_{x_0}\left(-\frac{dE}{dx_0}\right)$. The pre-factor $(\lambda_{\mathrm{m}}/a_{h\mathrm{BN}})$ accounts for the ratio between moiré superlattice periodicity, in units of which the potential of Eq. (S2) is written, and the atomic lattice periodicity. Using this approach, in the following, we present the derivations for the static friction of the aligned polygonal flakes considered in this study, that demonstrate non-zero global scaling.

4.1. Aligned rectangular and square flakes

Consider a rectangular flake with lateral dimensions of L_x and L_y, and geometric center positioned at (x_0, y_0) in the reference frame of moiré superlattice and stacked in the aligned configuration. The total potential experienced by the flake can be calculated as:

$$E^{\mathrm{rec}}(L_x, L_y; x_0, y_0) = \int_{x_0-\frac{L_x}{2}}^{x_0+\frac{L_x}{2}}\int_{y_0-\frac{L_y}{2}}^{y_0+\frac{L_y}{2}} U(x,y)dxdy =$$

$$= -\frac{\sqrt{3}\lambda_{\mathrm{m}}U_0}{9\pi^2}\left(4\lambda_{\mathrm{m}}\cos\frac{2\pi x_0}{\sqrt{3}\lambda_{\mathrm{m}}}\sin\frac{\pi L_x}{\sqrt{3}\lambda_{\mathrm{m}}}\cos\frac{2\pi y_0}{\lambda_{\mathrm{m}}}\sin\frac{\pi L_y}{\lambda_{\mathrm{m}}} + \pi L_y\cos\frac{4\pi x_0}{\sqrt{3}\lambda_{\mathrm{m}}}\sin\frac{2\pi L_x}{\sqrt{3}\lambda_{\mathrm{m}}}\right). \quad \text{(S3)}$$

The x component of the lateral force required to overcome this flake-substrate interaction is:

$$F_{x_0}^{\mathrm{rec}}(L_x, L_y; x_0, y_0) = \frac{\partial E^{\mathrm{rec}}(A; x_0, y_0)}{\partial x_0} =$$

$$= \frac{4}{9}U_0\left(\frac{2\lambda_{\mathrm{m}}}{\pi}\sin\frac{2\pi x_0}{\sqrt{3}\lambda_{\mathrm{m}}}\sin\frac{\pi L_x}{\sqrt{3}\lambda_{\mathrm{m}}}\cos\frac{2\pi y_0}{\lambda_{\mathrm{m}}}\sin\frac{\pi L_y}{\lambda_{\mathrm{m}}} + L_y\sin\frac{4\pi x_0}{\sqrt{3}\lambda_{\mathrm{m}}}\sin\frac{2\pi L_x}{\sqrt{3}\lambda_{\mathrm{m}}}\right). \quad \text{(S4)}$$

When $L_y \gg \lambda_{\rm m}$, the second the term on the right-hand side dominates this expression, yielding the following approximation for the static friction force:

$$F^{\rm rec}_{\rm s,rigid}(L_x,L_y) = \left(\frac{\lambda_{\rm m}}{a_{h\rm BN}}\right)\max_{x_0}\left(-F^{\rm rec}_{x_0}(L_x,L_y;x_0)\right) \approx \frac{4}{9}\frac{\lambda_{\rm m}L_yU_0}{a_{h\rm BN}}\left|\sin\left(\frac{2\pi L_x}{\sqrt{3}\lambda_{\rm m}}\right)\right|. \tag{S5}$$

For rectangular flakes of length-to-width ratio of $L_x/L_y = \sqrt{3}$, considered in Fig. S3(a)-(b) and Fig. S4(a)-(b), the upper envelope is therefore:

$$F^{\rm up,rec}_{\rm s,rigid}(A) \approx \frac{4}{9}\frac{\lambda_{\rm m}L_yU_0}{a_{h\rm BN}} = \frac{4}{9\sqrt[4]{3}}\frac{\lambda_{\rm m}U_0}{a_{h\rm BN}}A^{\frac{1}{2}}. \tag{S6}$$

Similarly, for square flakes $(L_x/L_y = 1)$, the upper envelope is:

$$F^{\rm up,sqr}_{\rm s,rigid}(A) = \frac{4}{9}\frac{\lambda_{\rm m}U_0}{a_{h\rm BN}}A^{\frac{1}{2}}. \tag{S7}$$

4.2. Aligned triangular flakes

Consider an equilateral triangular flake with size length of L_0 and geometric center positioned at (x_0, y_0) in the reference frame of moiré superlattice and stacked in the aligned configuration. The total potential experienced by the flake can be calculated as:

$$E^{\rm tri}(L_0;x_0,y_0) = \int_{x_0-\frac{\sqrt{3}L_0}{6}}^{x_0+\frac{\sqrt{3}L_0}{3}}\int_{y_0-\frac{L_0}{3}+\frac{\sqrt{3}}{3}(x-x_0)}^{y_0+\frac{L_0}{3}-\frac{\sqrt{3}}{3}(x-x_0)} U(x,y)dxdy =$$

$$= \frac{\sqrt{3}\lambda_{\rm m}U_0}{36\pi^2}\left(2\pi L_0\left(\sin\frac{4\pi}{\sqrt{3}\lambda_{\rm m}}\left(x_0-\frac{\sqrt{3}L_0}{6}\right) - 2\cos\frac{2\pi y_0}{\lambda_{\rm m}}\sin\frac{2\pi}{\sqrt{3}\lambda_{\rm m}}\left(\frac{\sqrt{3}L_0}{3}+x_0\right)\right) + \lambda_{\rm m}\left(\cos\frac{4\pi}{\sqrt{3}\lambda_{\rm m}}\left(x_0+\frac{\sqrt{3}L_0}{3}\right) - \cos\frac{4\pi}{\sqrt{3}\lambda_{\rm m}}\left(x_0-\frac{\sqrt{3}L_0}{6}\right) + 4\cos\frac{2\pi y_0}{\lambda_{\rm m}}\sin\frac{\pi L_0}{\lambda_{\rm m}}\sin\frac{2\pi}{\sqrt{3}\lambda_{\rm m}}\left(x_0-\frac{\sqrt{3}L_0}{6}\right)\right)\right). \tag{S8}$$

The x component of the lateral force required to overcome this flake-substrate interaction is:

$$F^{\rm tri}_{x_0}(L_0;x_0,y_0) = \frac{\partial E^{\rm tri}(L_0;x_0,y_0)}{\partial x_0} = \frac{U_0}{9\pi}\left(2\pi L_0\left(\cos\frac{4\pi}{\sqrt{3}\lambda_{\rm m}}\left(x_0-\frac{\sqrt{3}L_0}{6}\right) - \cos\frac{2\pi y_0}{\lambda_{\rm m}}\cos\frac{2\pi}{\sqrt{3}\lambda_{\rm m}}\left(\frac{\sqrt{3}L_0}{3}+x_0\right)\right) + \lambda_{\rm m}\left(\sin\frac{4\pi}{\sqrt{3}\lambda_{\rm m}}\left(x_0-\frac{\sqrt{3}L_0}{6}\right) - \sin\frac{4\pi}{\sqrt{3}\lambda_{\rm m}}\left(x_0+\frac{\sqrt{3}L_0}{3}\right) + 2\cos\frac{2\pi y_0}{\lambda_{\rm m}}\sin\frac{\pi L_0}{\lambda_{\rm m}}\cos\frac{2\pi}{\sqrt{3}\lambda_{\rm m}}\left(x_0-\frac{\sqrt{3}L_0}{6}\right)\right)\right) \tag{S9}$$

Since an analytical expression for the maximal value of $F^{\rm tri}_{x_0}(L_0;x_0)$ cannot be found, the static friction of triangular flakes, $F^{\rm tri}_{\rm s,rigid}$, is evaluated numerically. Keeping only the leading term in L_0 we obtain:

$$F_{\text{s,rigid}}^{\text{tri}}(L_0; x_0, y_0) \approx \frac{2\lambda_{\text{m}} U_0 L_0}{9a_{h\text{BN}}} \max_{x_0} \left[-\left(\cos\frac{4\pi}{\sqrt{3}\lambda_{\text{m}}}\left(x_0 - \frac{\sqrt{3}L_0}{6}\right) - \cos\frac{2\pi y_0}{\lambda_{\text{m}}} \cos\frac{2\pi}{\sqrt{3}\lambda_{\text{m}}}\left(\frac{\sqrt{3}L_0}{3} + x_0\right)\right)\right] \quad \text{(S10)}$$

From Eq. (S10) we can estimate the upper envelope as:

$$F_{\text{s,rigid}}^{\text{up,tri}}(A) \approx \frac{4}{9}\frac{\lambda_{\text{m}} U_0 L_0}{a_{h\text{BN}}} = \frac{8}{9\sqrt[4]{3}}\frac{\lambda_{\text{m}} U_0}{a_{h\text{BN}}} A^{\frac{1}{2}} \quad \text{(S11)}$$

4.3. Hexagonal flake

Consider an equilateral hexagonal flake with side length of L_0 and geometric center positioned at (x_0, y_0) in the reference frame of the moiré superlattice and stacked in the aligned configuration. The total potential experienced by the flake can be calculated as:

$$E^{\text{hex}}(L_0; x_0, y_0) = \int_{x_0}^{x_0+\frac{\sqrt{3}L_0}{2}} \int_{y_0-\frac{L_0}{2}-\frac{\sqrt{3}}{3}(x-x_0)}^{y_0+\frac{L_0}{2}+\frac{\sqrt{3}}{3}(x-x_0)} U(x,y)dxdy + \int_{x_0+\frac{\sqrt{3}L_0}{2}}^{x_0+\sqrt{3}L_0} \int_{y_0-\frac{3L_0}{2}+\frac{\sqrt{3}}{3}(x-x_0)}^{y_0+\frac{3L_0}{2}-\frac{\sqrt{3}}{3}(x-x_0)} U(x,y)dxdy =$$

$$= -\frac{\sqrt{3}\lambda_{\text{m}}}{9\pi^2} U_0 \left(\lambda_{\text{m}} \sin^2\frac{\pi L_0}{\lambda_{\text{m}}} + \pi L_0 \sin\frac{2\pi L_0}{\lambda_{\text{m}}}\right)\left(2\cos\frac{2\pi}{\sqrt{3}\lambda_{\text{m}}}\left(\frac{\sqrt{3}}{2}L_0 + x_0\right)\cos\frac{2\pi y_0}{\lambda_{\text{m}}} + \cos\frac{4\pi}{\sqrt{3}\lambda_{\text{m}}}\left(x_0 + \frac{\sqrt{3}L_0}{2}\right)\right) \quad \text{(S12)}$$

The x component of the lateral force required to overcome this flake-substrate interaction is:

$$F^{\text{hex}}(L_0; x_0, y_0) = \frac{4}{9\pi} U_0 \left(\lambda_{\text{m}} \sin^2\frac{\pi L_0}{\lambda_{\text{m}}} + \pi L_0 \sin\frac{2\pi L_0}{\lambda_{\text{m}}}\right)\left(\sin\frac{2\pi}{\sqrt{3}\lambda_{\text{m}}}\left(\frac{\sqrt{3}}{2}L_0 + x_0\right)\cos\frac{2\pi y_0}{\lambda_{\text{m}}} + \sin\frac{4\pi}{\sqrt{3}\lambda_{\text{m}}}\left(x_0 + \frac{\sqrt{3}L_0}{2}\right)\right). \quad \text{(S13)}$$

When $L_0 \gg \lambda_{\text{m}}$, $F^{\text{hex}}(L_0; x_0, y_0 = 0)$ can be approximated as:

$$F^{\text{hex}}(L_0; x_0, y_0 = 0) \approx \frac{4}{9} U_0 L_0 \sin\frac{2\pi L_0}{\lambda_{\text{m}}}\left(\sin\frac{2\pi}{\sqrt{3}\lambda_{\text{m}}}\left(\frac{\sqrt{3}}{2}L_0 + x_0\right) + \sin\frac{4\pi}{\sqrt{3}\lambda_{\text{m}}}\left(x_0 + \frac{\sqrt{3}L_0}{2}\right)\right). \quad \text{(S14)}$$

The static friction force of the hexagonal flake along the scanline $y_0 = 0$ can be calculated as:

$$F_{\text{s,rigid}}^{\text{hex}}(L_0) = \left(\frac{\lambda_{\text{m}}}{a_{h\text{BN}}}\right) \max_{x_0}\left(-F^{\text{hex}}(L_0; x_0)\right) \approx \frac{(3+\sqrt{33})\sqrt{30+2\sqrt{33}}}{72}\frac{\lambda_{\text{m}} U_0 L_0}{a_{h\text{BN}}}\left|\sin\frac{2\pi L_0}{\lambda_{\text{m}}}\right| \quad \text{(S15)}$$

Correspondingly, the upper envelope is given by:

$$F_{\text{s,rigid}}^{\text{up,hex}}(A) \approx \frac{(3+\sqrt{33})\sqrt{30+2\sqrt{33}}}{72}\frac{\lambda_{\text{m}}}{a_{h\text{BN}}} L_0 U_0 = \frac{(\sqrt{3}+\sqrt{11})\sqrt{5\sqrt{3}+\sqrt{11}}}{36}\frac{\lambda_{\text{m}} U_0}{a_{h\text{BN}}} A^{\frac{1}{2}} \quad \text{(S16)}$$

5. Frictional Dissipation Analysis

5.1. Determination of the dominant frictional interface

In the main text, we have stated that frictional energy dissipation occurs at the heterogeneous interface between the bottom graphene slider layer and the top *h*-BN substrate surface. The generated frictional heat is then assumed to transfer to the adjacent layers, where it is dissipated via damped dynamics in our simulations.

To verify that this is indeed the case, we calculated the time averaged kinetic energy map of the bottom graphene flake layer (l_3, see Fig. 1 of the main text) and the top *h*-BN substrate layer (l_4). As shown in Fig. S7, the obtained kinetic energy distribution maps correspond well with the power dissipation distribution maps at layer l_2, presented in Fig. 3 of the main text. This demonstrates that heat generated at the sliding interface propagates to adjacent layers where it is dissipated.

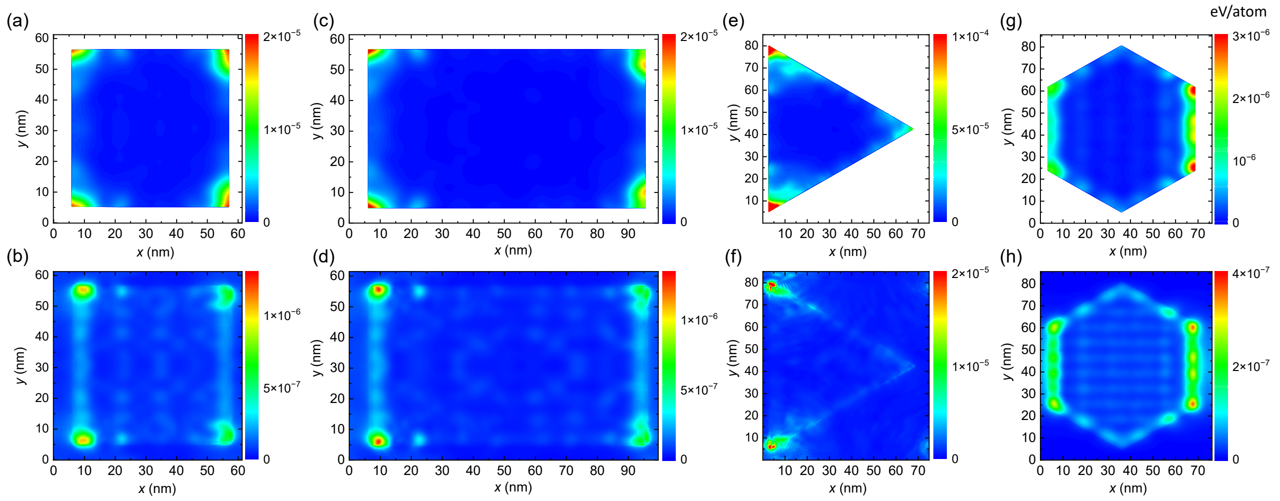

Figure S7. Time averaged kinetic energy distribution map per atom in (a) the bottom graphene flake layer (l_3) and (b) the top *h*-BN substrate layer (l_4) for a square shaped slider. (c) and (d) same as (a) and (b) for a rectangular slider. (e) and (f) same as (a) and (b) for a triangular slider. (g) and (h) same as (a) and (b) for a hexagonal slider. The flake dimensions are the same as those presented in Fig. 3 of the main text.

5.2. Calculation of energy dissipation power and analysis of dissipation routes

At steady state, the system achieves a general power balance $(p_{\text{in}} = p_{\text{diss}})$ between the input from the moving stage (p_{in}) and the total dissipation through viscous damping ($p_{\text{diss}} = F_{\text{k}} \cdot v_0$). At zero temperature, where thermal noise is absent, the total dissipation power p_{diss} can be calculated according to the viscous damping scheme in Eq. (S1) as follows:

$$p_{\text{diss}} = p_{\text{diss}}^{l_2} + p_{\text{diss}}^{l_5},$$

$$p_{\text{diss}}^{l_2} = \sum_i^{N_{l_2}} m_{\text{C}}\,\eta \left(\left\langle \left[v_x^{i,l_2}(t) - v_0\right]^2 \right\rangle + \sum_{\alpha=y,z} \langle v_\alpha^{i,l_2}(t)^2 \rangle\right), \tag{S16}$$

$$p_{\text{diss}}^{l_5} = m^i \eta \sum_i^{N_{l_5}} \sum_{\alpha=x,y,z} \langle v_\alpha^{i,l_5}(t)^2 \rangle, \quad m^i = m_{\text{B}} \text{ or } m_{\text{N}},$$

where $N_{l_{k=2,5}}$ is the number of atoms in layer l_k and $\langle\cdot\rangle$ denotes a steady-state temporal average.

To elucidate the dissipation routes, we analyze the dissipation components in each Cartesian direction and their sums in each damped layer. We find that the damped finite-sized graphene flake layer (l_2) contributes ~90% of the total energy dissipated. Dissipation is found to be dominated by in-plane motion, along the sliding (x) direction (see Fig. S8(a)-(b)). Based on this we opt to present in the main text only the results of energy dissipation distribution in the damped graphene layer (l_2).

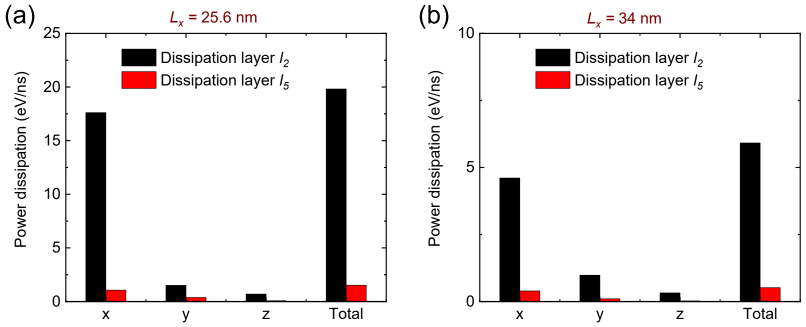

Figure S8. Time averaged energy dissipation power obtained for rectangular flakes of lengths (a) $L_x = 25.6$ nm and (b) $L_x = 34$ nm and an aspect ratio of $\sqrt{3}$, calculated at the middle graphene (black) and middle *h*-BN (red) layers, along the three cartesian directions and compared to the total dissipated power.

5.3. Edge energy dissipation power cross-sections

In terms of energy dissipation maps, slider corners are defined as the regions near the geometric corners where energy dissipation is significantly higher than the background inner surface contribution. Following this definition, we find the corners to be of ~10-20 nm. This is demonstrated in Fig. S9, where edge cross-sections of the dissipation map appearing in Fig. 3b of the main text, are presented.

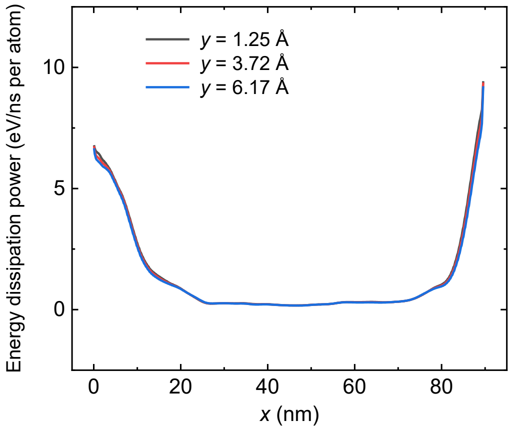

Figure S9. Energy dissipation power cross-sections taken near the bottom edge of the 2D heat-map presented in Fig. 3b of the main text.

5.4. Energy dissipation analysis of a laterally periodic heterogeneous graphene/*h*-BN interface

To complement the energy dissipation analysis in Fig. 3 of the main text, we present herein the dissipation power analysis for the laterally periodic graphene/*h*-BN interface. As shown in Fig. S10, the dissipation power contribution of the interior surface region is negligible compared to that of the corner and side counterparts (see Fig. S8, note the different y-axis values) and is mainly associated with vertical atomic motion in the graphene slider,[9] rather than in plane motion dissipation observed at the circumference of finite flakes.

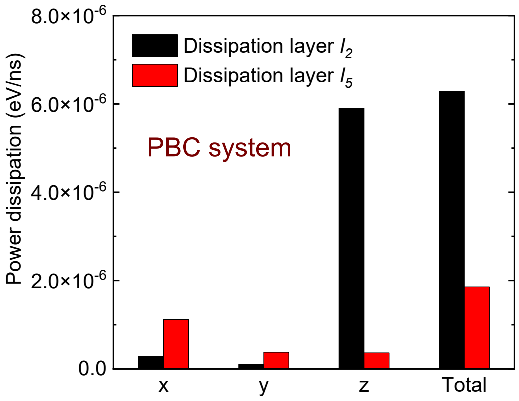

Figure S10. Time averaged energy dissipation power obtained for the laterally periodic graphene/*h*-BN interface calculated at the middle graphene (black) and middle *h*-BN (red) layers, along the three Cartesian directions and compared to the total dissipated power. The lateral dimensions of the supercell are taken to be $47.73 \times 27.55\ nm^2$.

5.5. Variation of energy dissipation power distribution with dimensions of rectangular flakes

To visualize the periodicity in the size scaling of the kinetic friction, we present the energy dissipation power distribution maps for rectangular flakes with varying aspect ratio by changing one of the side lengths while fixing the other. As shown in Fig. S11(a)-(c), varying the dimension perpendicular to the sliding direction results in a clear periodicity of the energy dissipation power map. Conversely, varying the flake dimension parallel to the sliding axis does not influence the dissipation pattern (see Fig. S11(d)-(f)). These results correspond well with the frictional behavior presented in Fig. 2(b) of the main text.

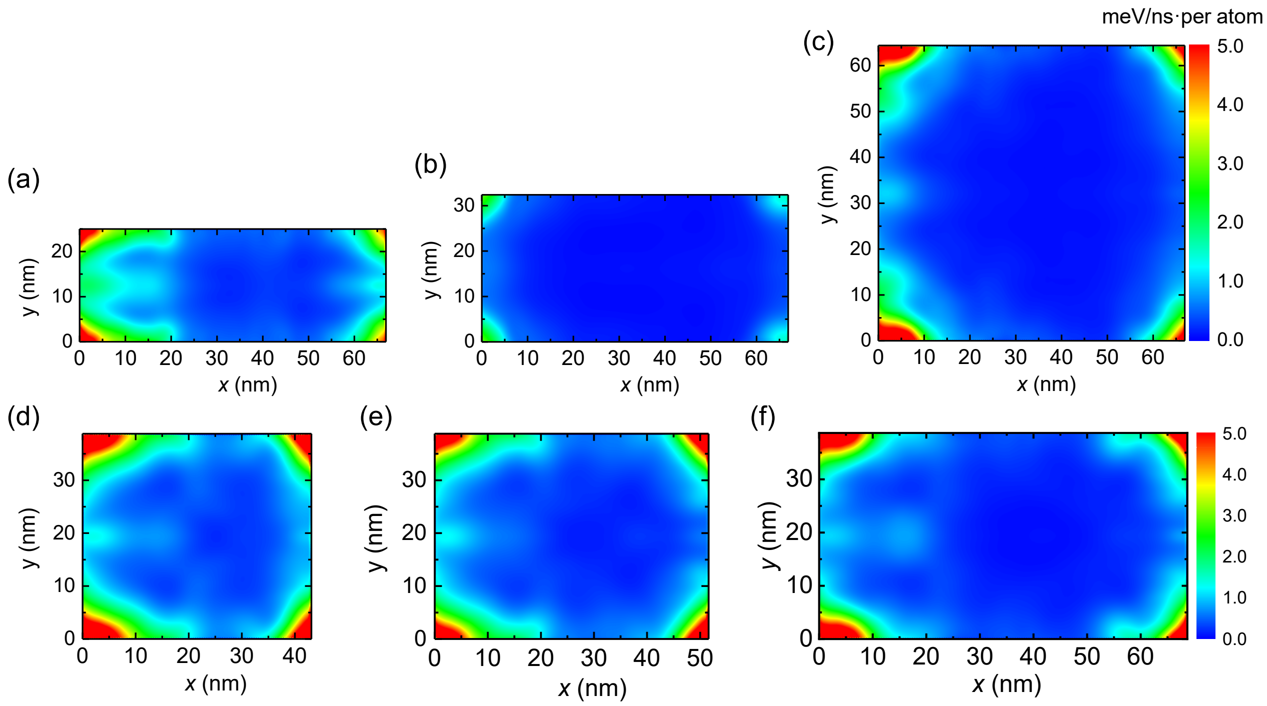

Figure S11. Time averaged power dissipation maps for rectangular flakes of different aspect ratios. (a)-(c) Energy dissipation power maps obtained by varying L_y, while fixing L_x. (d)-(f) same as (a)-(c) but for varying L_x at fixed L_y. Panels (a), (c), (d), (f) and panels (b), (e) correspond to the local friction maxima and minima in Fig. 2(b) of the main text, respectively.

5.6. Effect of slider thickness on energy dissipation distribution

To demonstrate the effect of slider thickness we repeated some of our simulations for a 6-layered square graphene slider of side-length of 25.6 nm. The power dissipation maps of the 4 damped layers are presented in Fig. S12, showing that the dissipated power reduces with vertical distance from the sliding interface, but remains localized at the flake corners.

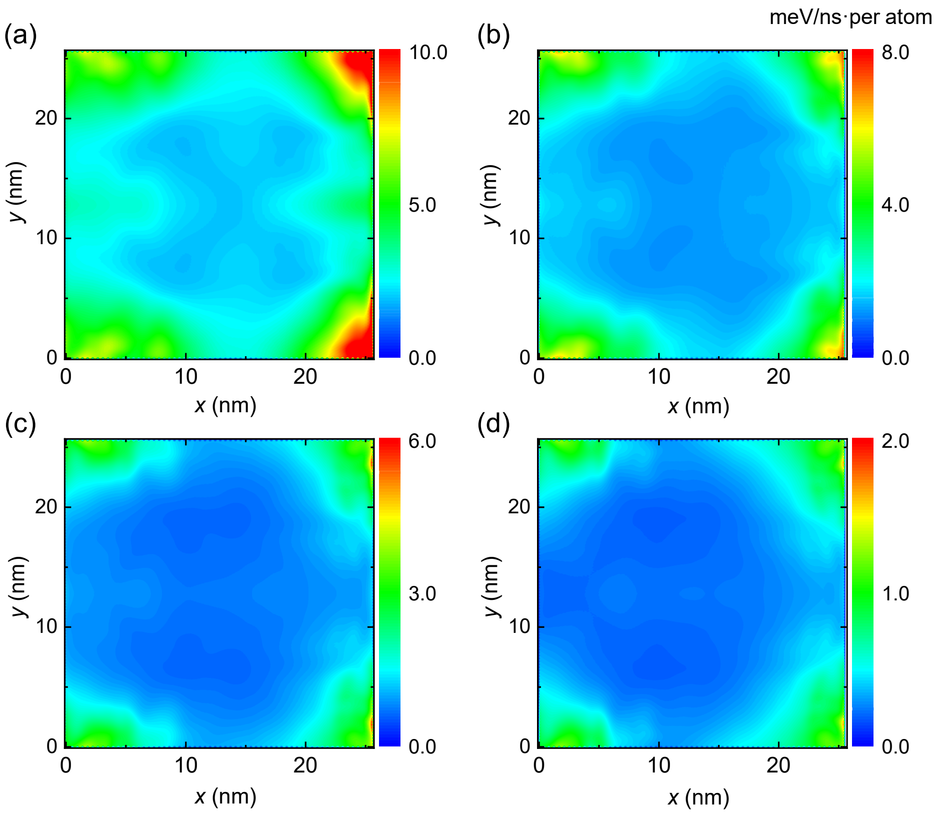

Figure S12. Time averaged power dissipation maps in the (a) second (adjacent to the bottom most graphene layer), (b) third, (c) fourth, and (d) fifth damped graphene layers of a 6-layer square flake slider of side length of 25.6 nm.

References:

(1) Gao, X.; Ouyang, W.; Hod, O.; Urbakh, M. Mechanisms of frictional energy dissipation at graphene grain boundaries. *Phys. Rev. B* **2021**, *103* (4), 045418

(2) Gao, X.; Ouyang, W.; Urbakh, M.; Hod, O. Superlubric polycrystalline graphene interfaces. *Nat. Commun.* **2021**, *12* (1), 5694

(3) Gao, X.; Urbakh, M.; Hod, O. Stick-Slip Dynamics of Moiré Superstructures in Polycrystalline 2D Material Interfaces. *Phys. Rev. Lett.* **2022**, *129* (27), 276101

(4) Brenner, D. W.; Shenderova, O. A.; Harrison, J. A.; Stuart, S. J.; Ni, B.; Sinnott, S. B. A second-generation reactive empirical bond order (REBO) potential energy expression for hydrocarbons. *J. Phys.: Condens. Matter* **2002**, *14* (4), 783-802

(5) Tersoff, J. New empirical approach for the structure and energy of covalent systems. *Phys. Rev. B* **1988**, *37* (12), 6991-7000

(6) Mandelli, D.; Ouyang, W.; Urbakh, M.; Hod, O. The Princess and the Nanoscale Pea: Long-Range Penetration of Surface Distortions into Layered Materials Stacks. *ACS Nano* **2019**, *13* (7), 7603-7609

(7) Ouyang, W. G.; Azuri, I.; Mandelli, D.; Tkatchenko, A.; Kronik, L.; Urbakh, M.; Hod, O. Mechanical and Tribological Properties of Layered Materials under High Pressure: Assessing the Importance of Many-Body Dispersion Effects. *J. Chem. Theory Comput.* **2020**, *16* (1), 666-676

(8) Ouyang, W.; Sofer, R.; Gao, X.; Hermann, J.; Tkatchenko, A.; Kronik, L.; Urbakh, M.; Hod, O. Anisotropic Interlayer Force Field for Transition Metal Dichalcogenides: The Case of Molybdenum Disulfide. *J. Chem. Theory Comput.* **2021**, *17* (11), 7237-7245

(9) Mandelli, D.; Ouyang, W.; Hod, O.; Urbakh, M. Negative Friction Coefficients in Superlubric Graphite-Hexagonal Boron Nitride Heterojunctions. *Phys. Rev. Lett.* **2019**, *122* (7), 076102

(10) Kolmogorov, A. N.; Crespi, V. H. Registry-dependent interlayer potential for graphitic systems. *Phys. Rev. B* **2005**, *71* (23), 235415

(11) Leven, I.; Azuri, I.; Kronik, L.; Hod, O. Inter-layer potential for hexagonal boron nitride. *J. Chem. Phys.* **2014**, *140* (10), 104106

(12) Leven, I.; Maaravi, T.; Azuri, I.; Kronik, L.; Hod, O. Interlayer Potential for Graphene/*h*-BN Heterostructures. *J. Chem. Theory Comput.* **2016**, *12* (6), 2896-2905

(13) Maaravi, T.; Leven, I.; Azuri, I.; Kronik, L.; Hod, O. Interlayer Potential for Homogeneous Graphene and Hexagonal Boron Nitride Systems: Reparametrization for Many-Body Dispersion Effects. *J. Phys. Chem. C* **2017**, *121* (41), 22826-22835

(14) Ouyang, W.; Mandelli, D.; Urbakh, M.; Hod, O. Nanoserpents: Graphene Nanoribbon Motion on Two-Dimensional Hexagonal Materials. *Nano Lett.* **2018**, *18* (9), 6009-6016

(15) Krukau, A. V.; Vydrov, O. A.; Izmaylov, A. F.; Scuseria, G. E. Influence of the exchange screening parameter on the performance of screened hybrid functionals. *J. Chem. Phys.* **2006**, *125* (22), 224106

(16) Tkatchenko, A.; DiStasio, R. A.; Car, R.; Scheffler, M. Accurate and Efficient Method for Many-Body van der Waals Interactions. *Phys. Rev. Lett.* **2012**, *108* (23), 236402

(17) Ouyang, W.; Azuri, I.; Mandelli, D.; Tkatchenko, A.; Kronik, L.; Urbakh, M.; Hod, O. Mechanical and Tribological Properties of Layered Materials under High Pressure: Assessing the Importance of Many-Body Dispersion Effects. *J Chem Theory Comput* **2020**, *16* (1), 666-676

(18) Bitzek, E.; Koskinen, P.; Gähler, F.; Moseler, M.; Gumbsch, P. Structural Relaxation Made

Simple. *Phys. Rev. Lett.* **2006**, *97* (17), 170201

(19) Guénolé, J.; Nöhring, W. G.; Vaid, A.; Houllé, F.; Xie, Z.; Prakash, A.; Bitzek, E. Assessment and optimization of the fast inertial relaxation engine (fire) for energy minimization in atomistic simulations and its implementation in lammps. *Comput. Mater. Sci.* **2020**, *175*, 109584

(20) Song, Y.; Gao, X.; Pawlak, R.; Huang, S.; Hinaut, A.; Glatzel, T.; Hod, O.; Urbakh, M.; Meyer, E. Non-Amontons frictional behaviors of grain boundaries at layered material interfaces. *Nat. Commun.* **2024**, *15* (1), 9487

(21) Plimpton, S. Fast Parallel Algorithms for Short-Range Molecular Dynamics. *J. Comput. Phys.* **1995**, *117* (1), 1-19

(22) Thompson, A. P.; Aktulga, H. M.; Berger, R.; Bolintineanu, D. S.; Brown, W. M.; Crozier, P. S.; in 't Veld, P. J.; Kohlmeyer, A.; Moore, S. G.; Nguyen, T. D.; et al. LAMMPS - a flexible simulation tool for particle-based materials modeling at the atomic, meso, and continuum scales. *Comput. Phys. Commun.* **2022**, *271*, 108171

(23) Yan, W.; Gao, X.; Ouyang, W.; Liu, Z.; Hod, O.; Urbakh, M. Shape-dependent friction scaling laws in twisted layered material interfaces. *J. Mech. Phys. Solids* **2024**, *185*, 105555
